# Vinyl-Functionalized Janus Ring Siloxane: Potential Precursors to Hybrid Functional Materials

**DOI:** 10.3390/ma14082014

**Published:** 2021-04-16

**Authors:** Thanawat Chaiprasert, Yujia Liu, Nobuhiro Takeda, Masafumi Unno

**Affiliations:** Department of Chemistry and Chemical Biology, Graduate School of Science and Technology, Gunma University, Kiryu 376-8515, Japan; t182a007@gunma-u.ac.jp (T.C.); yliu@gunma-u.ac.jp (Y.L.); ntakeda@gunma-u.ac.jp (N.T.)

**Keywords:** Janus ring, silsesquioxane, hybrid organic-inorganic, vinyl monomer, Piers–Rubinsztajn reaction, cyclotetrasiloxane

## Abstract

A vinyl-functionalized all-*cis*-tetrasiloxycyclotetrasiloxane [ViSi(OSiMe_2_H)O]_4_ (Vi = vinyl group) Janus precursor was prepared from potassium cyclotetrasiloxane silanolate. The Janus precursor was selectively modified at its dimethylhydrosilyl groups [–SiMe_2_H] via the Piers–Rubinsztajn reaction to obtain a family of new tetravinyl-substituted Janus rings [ViSi(OR’)O]_4_ containing various functional groups in moderate yields. Remarkably, the tetravinyl groups on the structure remained intact after modification by the Piers–Rubinsztajn reaction. Since these synthesized compounds possess multiple functional groups (up to eight per molecule), they are potential precursors for advanced hybrid organic-inorganic functional materials.

## 1. Introduction

Hybrid organic-inorganic silsesquioxane precursors play an important role in the development of new materials and have a high potential for industrial applications [1,2,3]. Among them, vinyl-functionalized silsesquioxanes are interesting precursors because the vinyl groups can be modified by various methods, e.g., C–C coupling reaction [4,5,6,7], cross-metathesis [8,9], hydrosilylation [10], thiol-ene reaction [11,12,13,14], and polymerization [15,16,17]. In contrast to silica compounds that have high crystallinity and poor solubility, silsesquioxane compounds typically have an adjustable solubility and good dispersion in organic solvents or organic materials [18,19,20]. This is a desirable characteristic for their use as a nanofiller to improve the thermal properties of materials instead of harmful transition metal compounds [21,22,23,24].

The cyclotetrasiloxane T_4_ ring, a silsesquioxane compound with a core structure containing four silicon and four oxygen atoms connected with adjustable organic substituents ((RSi(OR’)O)_4_, shows promising characteristics like high thermal durability and a high refractive index with good solubility [25,26,27,28]. Furthermore, there are four possible isomeric structures of cyclotetrasiloxanes with two different substituents. Among them, all-*cis*-cyclotetrasiloxanes can be recognized as Janus molecules [29] because they have two different faces [30,31]. These Janus compounds have been used in the synthesis of well-defined nano-precursors [32,33,34,35], cubic silsesquioxanes T_8_ [30,36,37,38], copolymers [39,40], cyclic polymers [41,42,43], highly porous materials [4,11,12,44], semiconducting materials [45], protective coating molecules [46], and catalysts [47,48,49].

**Scheme 1:**
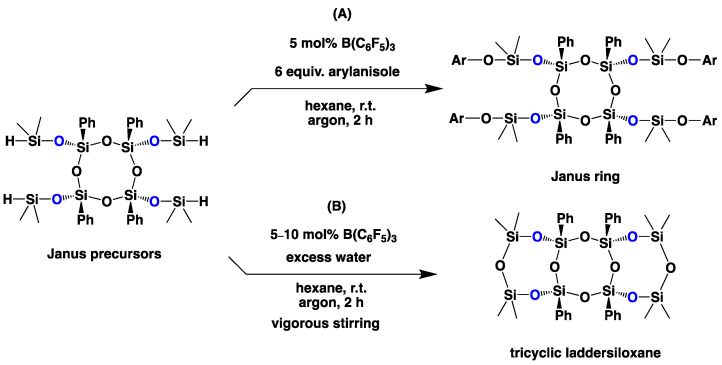
Previous work: (**A**) The synthesis of the Janus ring [PhSi(OSiMe_2_OR)O]_4_ (R = 4-methylphenyl, phenyl, 4-bromophenyl, 4-chlorophenyl, 4-(chloromethyl)phenyl, and 4-allylphenyl) by the Piers–Rubinsztajn reaction. (**B**) The synthesis of a tricyclic laddersiloxane by the intramolecular cyclization mediated by B(C_6_F_5_)_3_.

Although the synthesis of all-*cis*-cyclotetrasiloxanes with various substituents has been previously reported by several groups [30,36,50,51,52,53,54,55,56,57,58,59], there are only a few reported examples of modification, further functionalization, or bond extension of these compounds in the literature. For example, Makarova et al. [60] successfully modified the different stereoisomers of [PhSi(OSiMe_2_H)O]_4_ by hydrosilylation with H_2_C=CH(CH_2_)_8_COOC_6_H_4_C_6_H_4_CN. Previously, Marciniec et al. [61] synthesized various silsesquioxanes and demonstrated a proficient selective cross-metathesis reaction catalyzed by the ruthenium-hydride complex [RuHCl(CO)(PCy_3_)_2_] between olefins and tetramethyltetravinylcyclotetrasiloxane D_4_, which has a cyclic ring structure similar to that of T_4_. In 2012, Panisch et al. [29] reported the functionalization of all-*cis*-cyclotetrasiloxanes via the Heck and Sonogashira coupling reactions. The Piers–Rubinsztajn reaction, an efficient route for constructing the C–O–Si or Si–O–Si bonds [40,45,62,63,64,65,66,67], can also be used for the modification of hydrosilanes and hydrosilyl-functionalized linear, hyperbranched, cage T_8_, and double-decker silsesquioxanes [40,45,62,63,64,66,68,69,70,71,72,73,74,75,76,77,78,79,80,81,82,83,84]. In the case of all-*cis*-cyclotetrasiloxanes, we recently synthesized and characterized Janus-type phenyl-substituted all-*cis*-cyclotetrasiloxanes, as shown in Scheme 1 [35]. The isomerization reaction was not observed when using the Piers–Rubinsztajn reaction. In the presence of excess water, [PhSi(OSiMe_2_H)O]_4_ underwent an intramolecular cyclization reaction to form a six- or eight-membered side ring [85].

In continuation of our previous studies, herein we report the synthesis of various vinyl-functionalized Janus-type all-*cis*-cyclotetrasiloxanes [ViSi(OSiMe_2_OR)O]_4_. Remarkably, only the hydrosilyl group (–SiMe_2_H) of the starting material [ViSi(OSiMe_2_H)O]_4_ was transformed selectively in the Piers–Rubinsztajn reaction, and the vinyl groups (Vi) remained unreacted after the reaction. Furthermore, these compounds have high functional densities because they have four or eight functional groups per molecule.

## 2. Materials and Methods

### 2.1. General

All reactions in this study were conducted under an argon atmosphere (G2 grade (purity > 99.9995%, JAPAN FINE PRODUCTS (JFP), Kawasaki, Kanagawa, Japan) and stirred using Magnetic stirrer (PTFE stirrer, football type, As one, Osaka, Japan). All substrates were purchased from Tokyo Chemical Industry Co., Ltd., (Kawaguchi, Saitama, Japan) and used as received. The Janus precursor [ViSi(OSiMe_2_H)O]_4_ and potassium all-*cis*-tetravinylcyclotetrasiloxanolate were stored under anhydrous and argon atmospheres. All solvents were distilled and stored on anhydrous molecular sieves (Wako Pure Chemical Industries, Ltd., Osaka, Japan). Catalyst B(C_6_F_5_)_3_ was stored under an argon atmosphere. LC-5000 recycle-type preparative liquid chromatography was performed using a combination of a JAIGEL 1HR + 2HR (20 mm × 600 mm) GPC column (Japan Analytical Industry Co., Ltd., Tokyo, Japan) (eluent: CHCl_3_,). Fourier-transform NMR spectra were obtained on a JEOL JNM-ECS 600 NMR spectrometer (JEOL Ltd., Akishima, Tokyo, Japan), ^1^H at 600 MHz, ^13^C at 150.91 MHz, and ^29^Si at 119.24 MHz). MALDI-TOF mass spectrometry was performed on a Shimadzu MALDI-TOF AXIMA^®^ instrument (Shimadzu Corporation, Kyoto, Japan). IR spectra were measured with a Shimadzu FTIR-8400S instrument (Shimadzu Corporation, Kyoto, Japan).

### 2.2. Synthesis of Potassium All-Cis-Tetravinylcyclotetrasiloxanolate

**Scheme 2:**
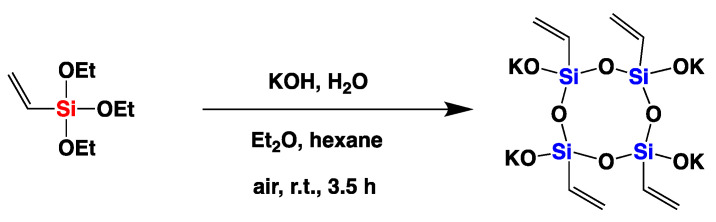
Synthesis of all-*cis*-tetravinylcyclotetrasiloxanolate by alkaline-direct condensation reaction.

As shown in Scheme 2, triethoxyvinylsilane (14.9 g, 88 mmol) was added dropwise to a round-bottom flask containing KOH (4.9 g, 88 mmol), water (1.6 g, 88 mmol), and hexane (90 mL) at room temperature. After stirring (RCT basic, IKA Japan K. K., Higashi-Osaka, Osaka, Japan) for 3.5 h, a white precipitate was formed. The precipitate was collected, washed with hexane, and dried using a high vacuum pump (G-20DA, ULVAC, Inc., Chigasaki, Kanagawa, Japan) for 1 day to yield potassium all-*cis*-tetravinylcyclotetrasiloxanolate as a white solid (5.00 g, 50% yield). Please note that this compound is highly hygroscopic and should be kept under an anhydrous atmosphere. In our study, it was used immediately after its preparation. Spectral data: ^29^Si NMR (methanol-d_4_) δ = −42.06 ppm.

### 2.3. Synthesis of Hydrido-Functionalized Janus Precursor [ViSi(OSiMe_2_H)O]_4_

**Scheme 3:**
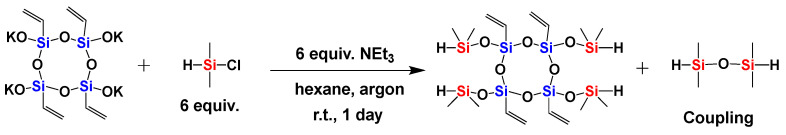
Synthesis of Janus precursor by condensation reaction.

As shown in Scheme 3, in a 250 mL two-necked round-bottom flask equipped with a magnetic stirrer, the white solid of potassium all-*cis*-tetravinylcyclotetrasiloxanolate (molecular weight 504.91, 5.00 g, 9.09 mmol) was added and evacuated for 1 day before use. Subsequently, the flask was refilled with argon. Then, anhydrous hexane (100 mL) and distilled NEt_3_ (7.6 mL, 54.54 mmol, 6 equiv.) were added to the reaction flask, and the mixture was vigorously stirred at −5 °C for 60 min. Next, SiMe_2_HCl (54.54 mmol, 6 equiv.) was added dropwise (1–2 drops per second) into the reaction flask via a glass syringe (Hamilton Company Inc., Reno, NV, USA). Water (200 mL) was then added to the reaction mixture, which was extracted with hexane (100 mL × 3). The combined organic layer was washed with water (200 mL × 3) and saturated NaCl solution once, dried over anhydrous Na_2_SO_4_, and concentrated using a high vacuum pump (G-20DA, ULVAC, Inc., Chigasaki, Kanagawa, Japan) for 1 day. After 1 day of evacuation, the pure product was obtained as a colorless liquid in 90% yield without purification.

Spectral data: ^1^H NMR of Janus precursor (CDCl_3_) δ = 0.26–0.26 ppm of CH_3_ (s); total H = 24H, 4.76–4.80 ppm of Si–H (m); 4H, 5.87–5.89 ppm for CH=CH_2_ (m); 8H, and 5.98–6.01 ppm (m) for CH=CH_2_; 4H), ^29^Si NMR of in CDCl_3_ (δ = −4.06 and −79.63 ppm).

### 2.4. Synthesis of Vinyl-Functionalized Janus Rings [ViSi(OSiMe_2_OR)O]_4_

In a 25 mL two-necked round-bottom flask equipped with a magnetic stirrer, Janus precursor [ViSi(OSiMe_2_H)O]_4_ (200 mg, 0.34 mmol) was mixed with a solution of aryl anisole (2.05 mmol, 6 equiv.) in anhydrous toluene (4 mL). Then, 5 mol% B(C_6_F_5_)_3_ (8.7 mg) was added to the reaction in an open system with an argon flow. After the addition of the catalyst, we observed that a gas was released spontaneously. The mixture was stirred at room temperature and subsequently quenched with water. Finally, the product was extracted using hexane, and the organic layer was washed with brine (CGC JAPAN CO., Ldt., Tokyo, Japan.) and dried over anhydrous Na_2_SO_4_. After solvent evaporation, the crude product was purified by GPC (CHCl_3_) (product yield and ^29^Si-NMR data are summarized in Table 1 and Supplementary Materials Table S1).

## 3. Results

The Janus precursor [ViSi(OSiMe_2_H)O]_4_ was prepared by the condensation of vinyl-functionalized potassium cyclotetrasiloxane silanolate all-*cis*-[ViSi(OK)O]_4_ with chlorodimethylsilane (Me_2_SiHCl) according to our previous report [27], as shown in Scheme 4. The reaction was conducted under argon atmosphere at low temperature (–5 °C) with the slow addition of Me_2_SiHCl in the presence of triethylamine (NEt_3_) to avoid side reactions such as acid-catalyzed isomerization and polymerization. These reaction conditions provided a 90% yield of the pure product ([ViSi(OSiMe_2_H)O]_4_), which was confirmed by Fourier transform infrared spectroscopy (FTIR) and nuclear magnetic resonance (NMR) spectroscopy (Supplementary Materials, Figures S1–S51).

Further, reaction screening of the Janus precursor [ViSi(OSiMe_2_H)O]_4_ was conducted. It revealed that the Pier–Rubinsztajn reaction conditions enabled selective transformation of dimethylhydrosilyl groups with various aryl anisoles (Table 1). Vinyl-functionalized Janus ring products Vi-JR-01 to Vi-JR-08 were successfully synthesized in the presence of 5 mol% B(C_6_F_5_)_3_ using an excess of the aryl anisole derivatives (1.5 equivalents per Vi) at room temperature for 1 day. Anhydrous toluene was used as the solvent because all the starting materials displayed good solubility in this solvent. It is worth noting that the reactions were conducted using an open system with an argon flow because the catalyst liberated flammable methane gas. Purification by gel permeation chromatography (GPC) provided the desired Janus rings in moderate yields (Table 1).

**Figure 1:**
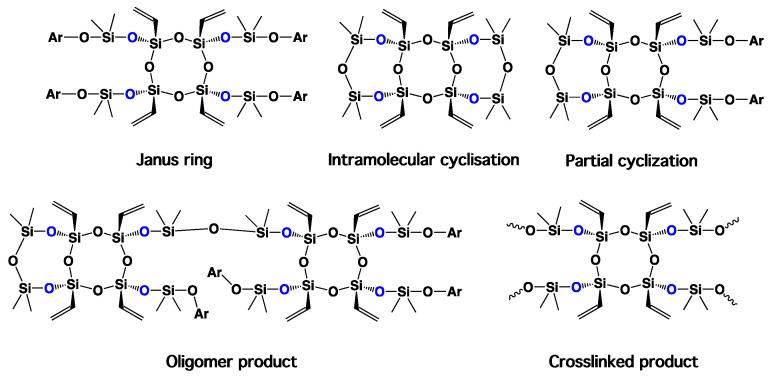
The structure of target product and possible by-products, including the partial intramolecular cyclization product, intramolecular cyclization product (tricyclic laddersiloxane), and crosslinked product.

**Scheme 5:**
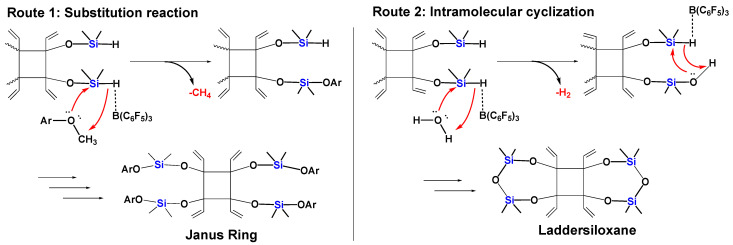
Proposed reaction mechanism [35].

In these reactions, the yields were affected by the purification methods because several byproducts formed as a result of partial intramolecular cyclization, intermolecular reaction, and polymerization, as shown in Figure 1. Owing to the interference of water or hydride migration, intramolecular cyclization took place competitively to partially form 6- or 8-membered cyclic or tricyclic laddersiloxanes as byproducts as shown in Scheme 5 [35,70,71,72,73,74,84,85].

^1^H, ^13^C, and ^29^Si NMR spectroscopy and matrix-assisted laser-desorption-ionization time-of-flight (MALDI-TOF) mass spectrometry were used to characterize the product structures. Similar results have been reported previously [35,59]; ^29^Si NMR spectra for Vi-JR-01 to Vi-JR-08 exhibited two peaks in the region from −11.7 to −12.3 ppm, corresponding to the D unit of Si in the –OSiMe_2_OAr arms, and in the region from −80.0 to −80.7 ppm, corresponding to the T unit Si atoms on the T_4_ ring of all-*cis*-cyclotetrasiloxanes. The disappearance of the signal at −4.08 ppm confirmed that all the Si–H in the starting material was transformed to –OSiMe_2_OAr. In the ^1^H NMR spectra, all the isolated products exhibited similar signals for the vinyl groups (CH=CH_2_) at 5.90–6.07 ppm, confirming that these groups were intact after the reaction. The lone signal of the T unit Si in the ^29^Si NMR spectrum of each product confirmed the conservation of the all-*cis* structure. All target Janus ring products are colorless viscous liquids with lower thermal properties (e.g., glass transition temperature or melting temperature) and lower crystallinity than previously reported tricyclic laddersiloxanes, double-decker, or octahedral oligomeric silsesquioxanes T_8_ [25,26,86,87,88,89,90,91].

Further investigations of the application of these vinyl-functionalized Janus rings as ion recognition molecules and porous materials are underway in our group. These products can be considered highly functionalized precursors because they have either four vinyl groups in each molecule (Vi-JR-01 to Vi-JR-03, and Vi-JR-08) or eight functional groups per unit (Vi-JR-04 to Vi-JR-07). Since they can be prepared more easily than octahedral oligomeric silsesquioxanes, vinyl-functionalized Janus rings can be used for the construction of advanced materials, such as well-defined cage silsesquioxanes, Janus-type nanomaterials, new polymers, and porous materials.

## 4. Conclusions

In this study, we successfully synthesized new vinyl-functionalized Janus-type all-*cis*-cyclotetrasiloxanes, [ViSi(OSiMe_2_OR)O]_4_ (R = 4-methylphenyl (Vi-JR-01), 2-methylphenyl (Vi-JR-02), phenyl (Vi-JR-03), 4-chlorophenyl (Vi-JR-04), 4-bromophenyl (Vi-JR-05), 4-iodophenyl (Vi-JR-06), 4-allylphenyl (Vi-JR-07), and naphthyl (Vi-JR-08)), by the Piers–Rubinsztajn reaction from the prepared Janus precursor. Currently, further investigations on the application of these compounds, e.g., as porous materials and ion-recognition-responsive materials, are underway in our group. Moreover, since these compounds have a high number of functional groups per unit, they are potential monomers of well-defined cage silsesquioxanes, Janus-type nanomolecules, and new polymers and porous materials.

## Figures and Tables

**Scheme 4:**
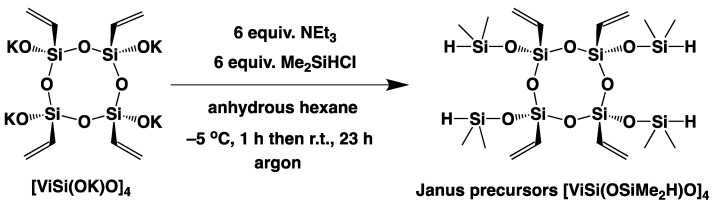
The synthesis of Janus precursors [ViSi(OSiMe_2_H)O]_4_ by the condensation of [ViSi(OK)O]_4_ with Me_2_SiHCl in the presence of NEt_3._

**Table 1:** The synthesis of Janus rings [ViSi(OSiMe_2_OR)O]_4_ Vi-JR-01 to Vi-JR-08 by Piers–Rubinsztajn reaction from the Janus precursor [ViSi(OSiMe_2_H)O]_4_ with an excess amount of aryl anisole.

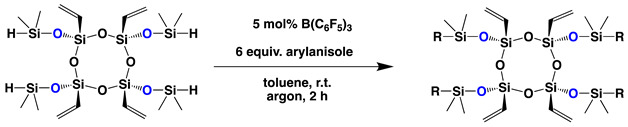			
Entry	Janus Rings	R	Isolated Yield ^1^ (%)
1	Vi-JR-01	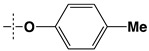	60
2	Vi-JR-02	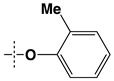	55
3	Vi-JR-03	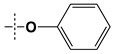	42
4	Vi-JR-04	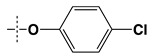	46
5	Vi-JR-05	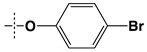	39
6	Vi-JR-06	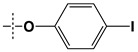	38
7	Vi-JR-07	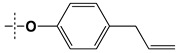	50
8	Vi-JR-08	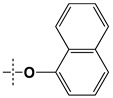	53

^1^ The yield was determined after purification using GPC (eluent = CHCl_3_). All products are colorless viscous liquids.

## Data Availability

Data is contained within the article.

## Supplementary Materials

The following are available online at https://www.mdpi.com/article/10.3390/ma14082014/s1, including ^1^H, ^13^C, and ^29^Si NMRs, MALDI-TOF-MS (Table S1, Figures S1 to S42), and FTIR spectra (Figures S43 to S51).
