# Supplementary material for: Vinyl-Functionalized Janus Ring Siloxane: Potential Precursors to Hybrid Functional Materials
Source: Materials (Basel). 2021 Apr 16;14(8):2014. doi: 10.3390/ma14082014 (PMC8073502; doi:10.3390/ma14082014)
Supplement: Supplementary file 1 [file materials-14-02014-s001.zip › materials-1145404-supplementary.pdf]

materials
MDPI

Supplementary

Vinyl-Functionalized Janus Ring Siloxane: Potential Precursors to Hybrid Functional Materials

Thanawat Chaiprasert, Yujia Liu, Nobuhiro Takeda and Masafumi Unno *

Department of Chemistry and Chemical Biology, Graduate School of Science and Technology, Gunma University, Kiryu 376–8515, Japan; t182a007@gunma-u.ac.jp (T.C.); yliu@gunma-u.ac.jp (Y.L.); ntakeda@gunma-u.ac.jp (N.T.)
* Correspondence: unno@gunma-u.ac.jp

Table S1. The summarization of ^{29}Si NMR of vinyl-functionalized Janus ring products.

Sample	^{29}Si NMR (119.24 MHz, $CDCl_3$)	
	D-unit Si (ppm)	T-unit Si (ppm)
Janus precursor	–4.08	–79.63
Vi-JR-01	−11.90	–80.69
Vi-JR-02	−12.29	–80.67
Vi-JR-03	−11.80	–80.69
Vi-JR-04	−11.21	–80.51
Vi-JR-05	−11.19	–80.51
Vi-JR-06	−10.68	–80.02
Vi-JR-07	−11.84	–80.68
Vi-JR-08	−11.18	–80.32

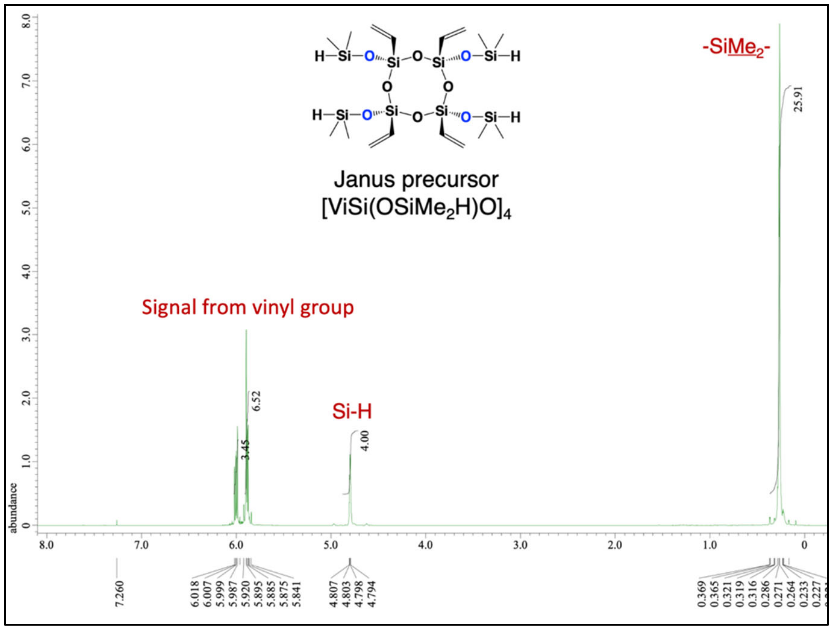

Figure S1. ^{1}H NMR (600.17 MHz, $CDCl_3$): δ 0.26 (s, 24H, Si<u>Me</u>$_2$), 4.79–4.80 (m, 4H, Si<u>H</u>), and 5.84–6.02 (m, 12H, C<u>H</u>=CH_2 and CH=C<u>H</u>$_2$ at vinyl group) ppm.

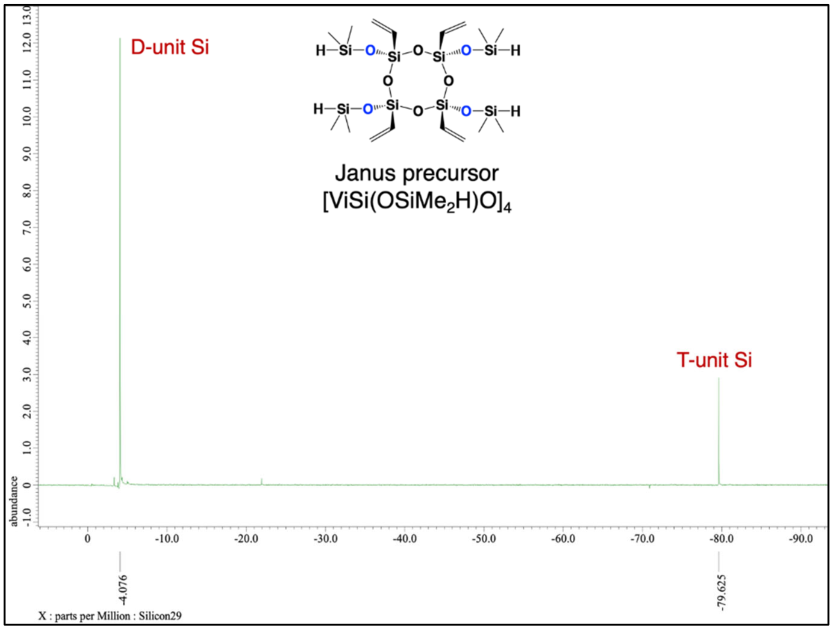

Figure S2. ^{29}Si NMR (119.24 MHz, $CDCl_3$): δ –4.08 ppm (D-unit Si) and –79.63 ppm (T-unit Si).

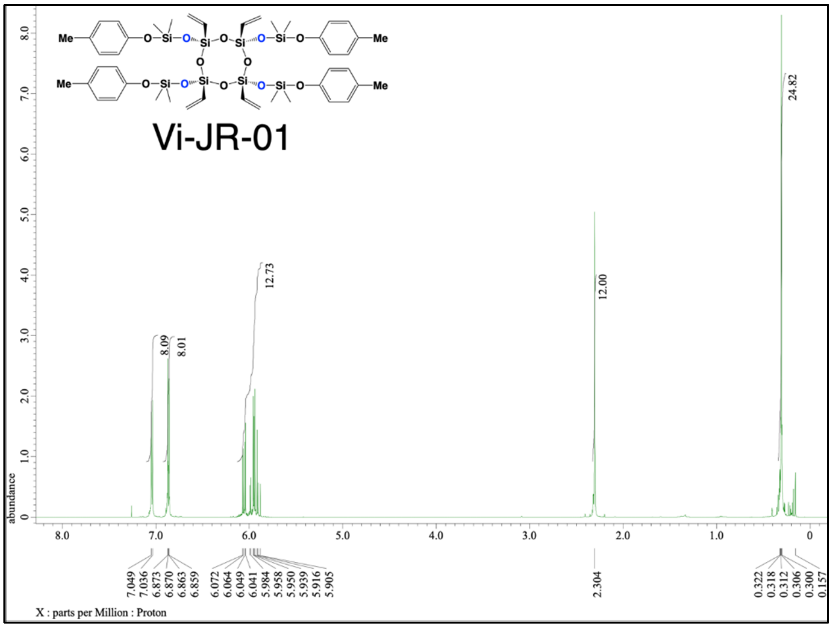

Figure S3. ^{1}H NMR (600.17 MHz, $CDCl_3$): δ 0.31 (s, 24H, Si<u>Me</u>$_2$), 2.30 (s, 12H, Ar–C<u>H</u>$_3$), 5.90–6.07 (m, 12H, C<u>H</u>=CH_2 and CH=C<u>H</u>$_2$ at vinyl group), and 6.85–7.05 ppm (m, 16H, Ar–H).

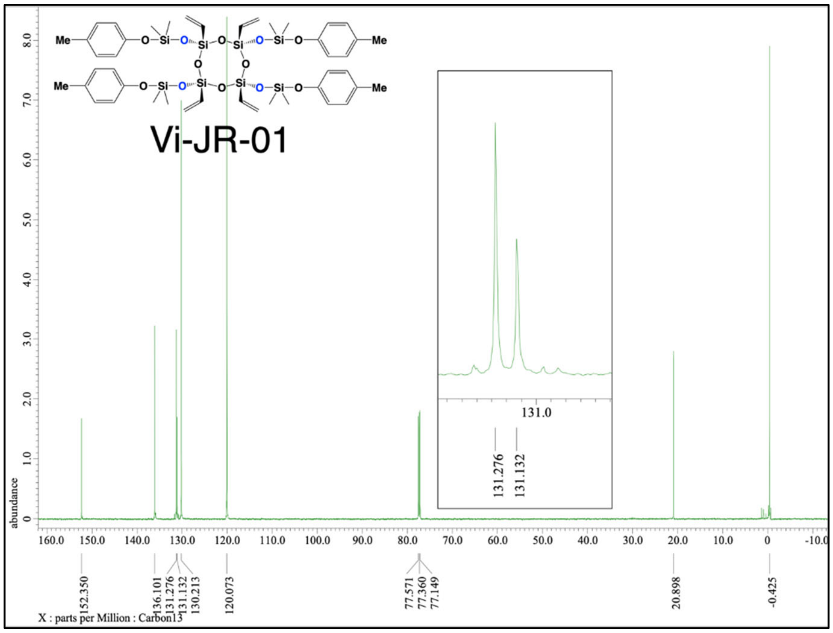

Figure S4. ^{13}C NMR (150.91 MHz, $CDCl_3$): δ –0.42, 20.90, 120.07, 130.21, 131.13, 131.28, 136.10, and 152.35 ppm.

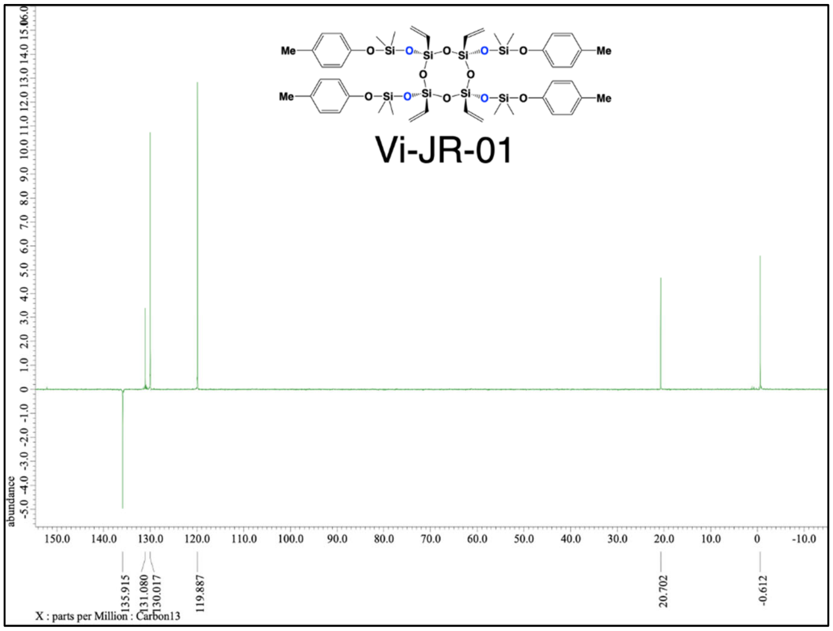

Figure S5. ^{13}C NMR (150.91 MHz, DEPT-135, $CDCl_3$): δ –0.61, 20.70, 119.89, 130.01, 131.08, and 135.92 ppm ($CH=CH_2$).

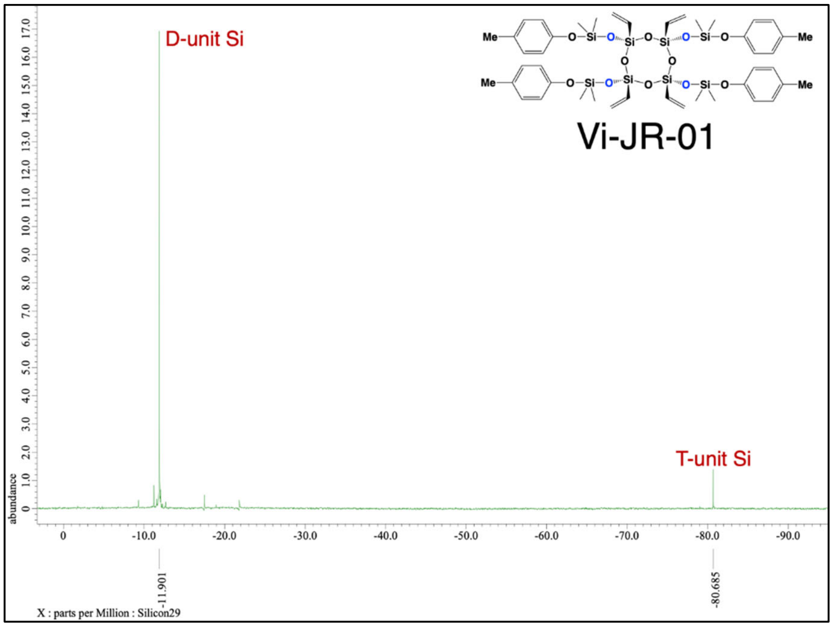

Figure S6. ²⁹Si NMR (119.24 MHz, $CDCl_3$): δ –11.90 ppm (D-unit Si) and –80.69 ppm (T-unit Si).

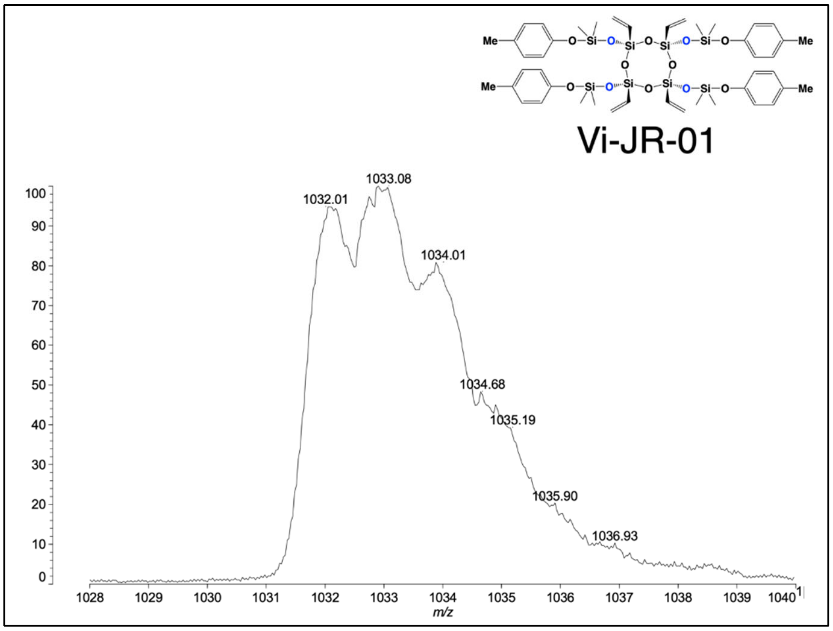

Figure S7. MALDI-TOF results of Vi-JR-01 (Calculated $[M + Na]^+$ = 1031.24).

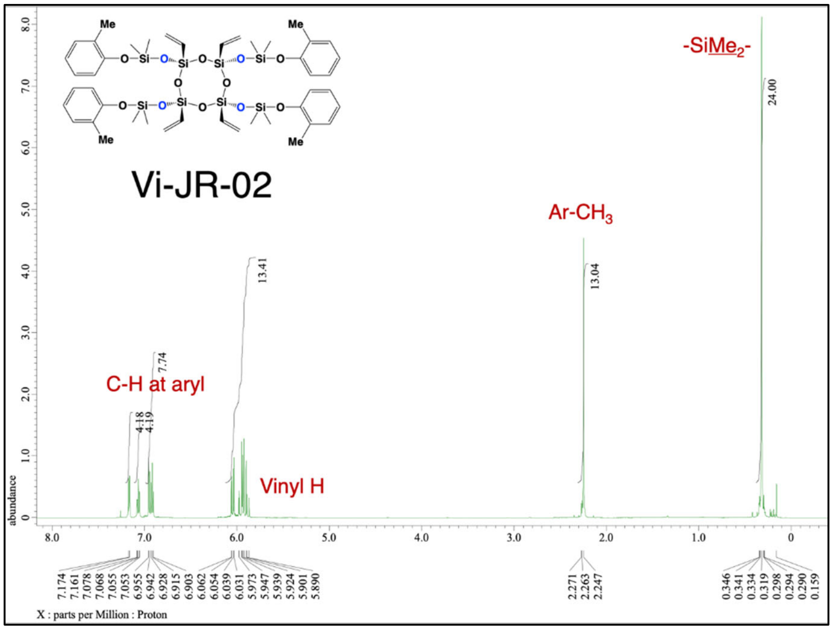

Figure S8. ^{1}H NMR (600.17 MHz, $CDCl_3$): δ 0.32 (s, 24H, Si<u>Me</u>$_2$), 2.26 (s, 12H, Ar–C<u>H</u>$_3$), 5.89–6.06 (m, 12H, C<u>H</u>=CH_2 and CH=C<u>H</u>$_2$ at vinyl group), and 6.90–7.17 ppm (m, 16H, Ar–H).

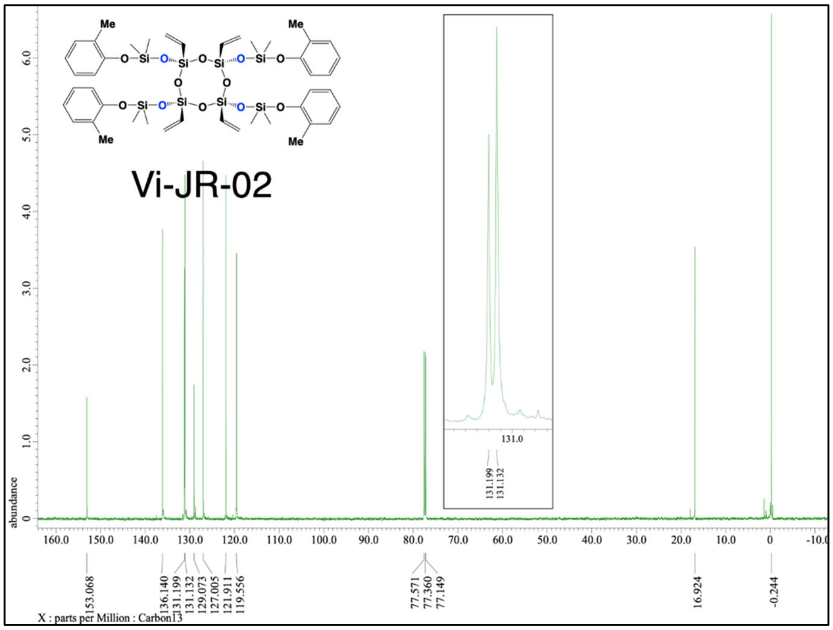

Figure S9. ^{13}C NMR (150.91 MHz, $CDCl_3$): δ –0.24, 16.92, 119.56, 121.91, 127.00, 129.07, 131.13, 131.19, 136.14, and 153.07 ppm.

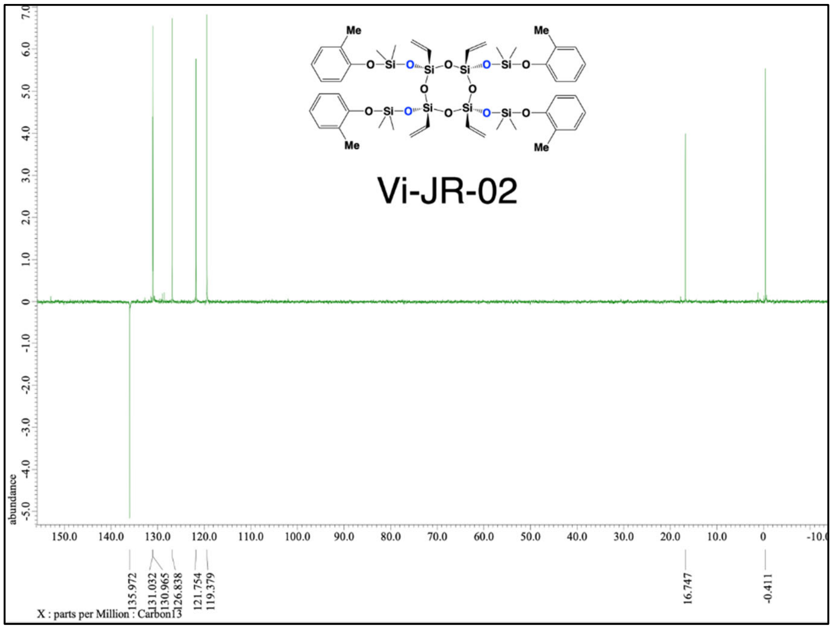

Figure S10. ^{13}C NMR (150.91 MHz, DEPT-135, $CDCl_3$): δ –0.41, 16.75, 119.37, 126.84, 130.97, 131.03, and 135.97 ppm ($CH=CH_2$).

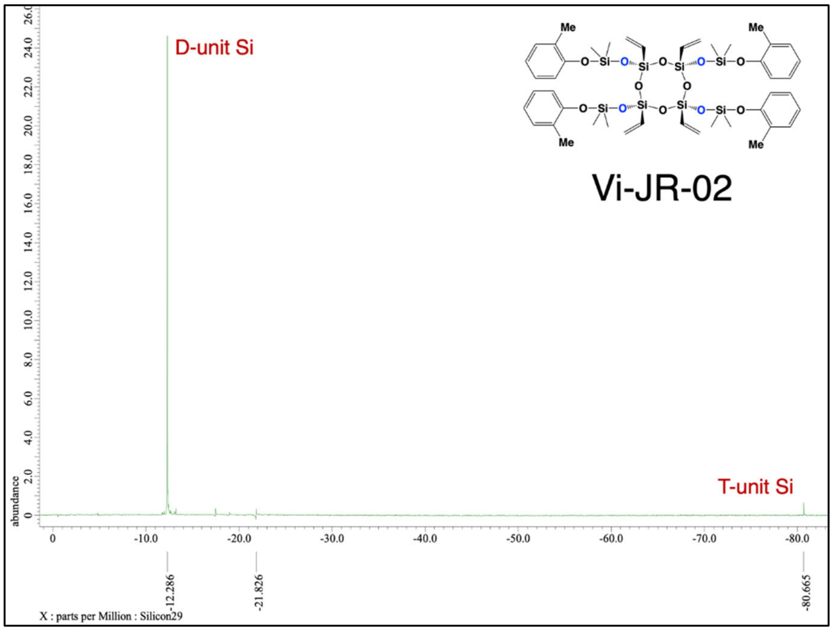

Figure S11. ^{29}Si NMR (119.24 MHz, $CDCl_3$): δ –12.29 ppm (D-unit Si) and –80.67 ppm (T-unit Si).

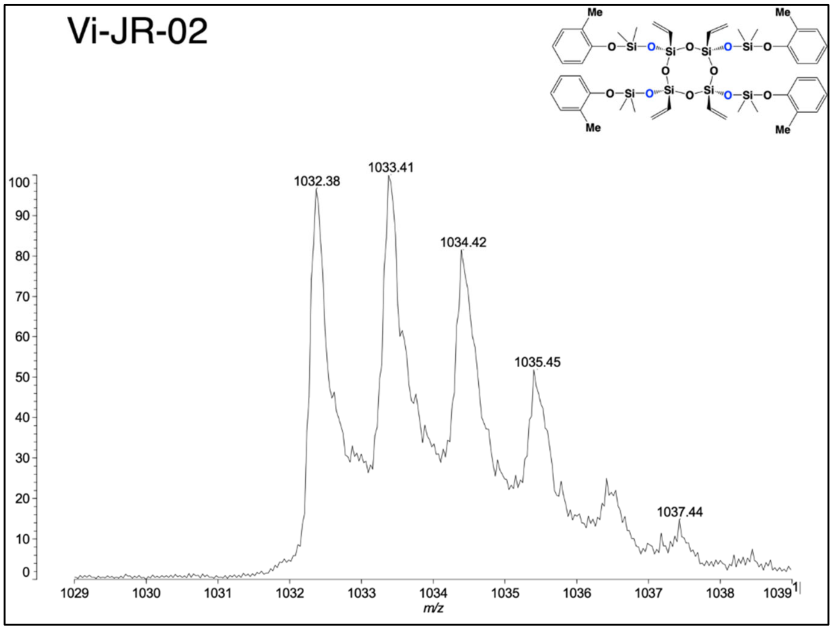

Figure S12. MALDI-TOF results of Vi-JR-02 (Calculated $[M + Na]^+$ = 1031.24).

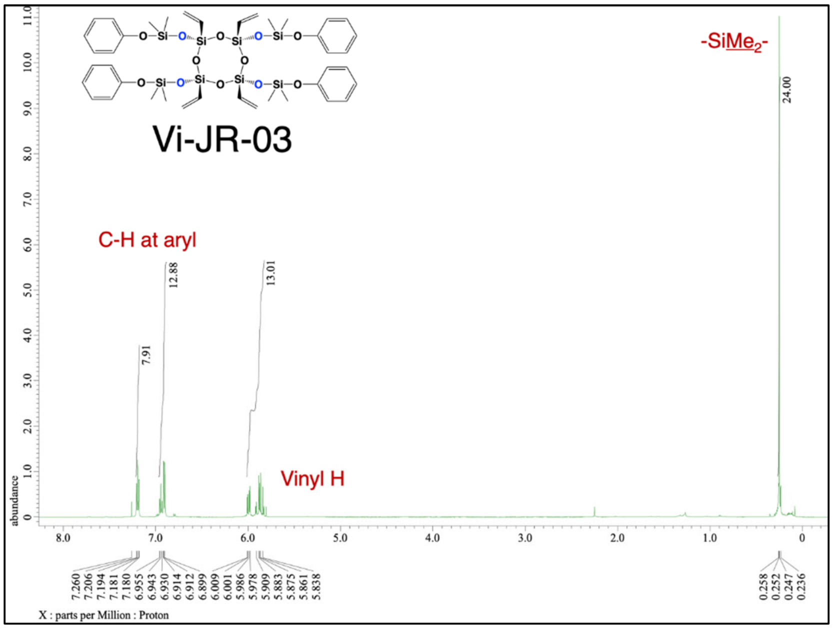

Figure S13. ^{1}H NMR (600.17 MHz, $CDCl_3$): δ 0.25 (s, 24H, Si<u>Me</u>$_2$), 5.84–6.00 (m, 12H, C<u>H</u>=CH_2 and CH=C<u>H</u>$_2$ at vinyl group), and 6.90–7.21 ppm (m, 16H, Ar–H).

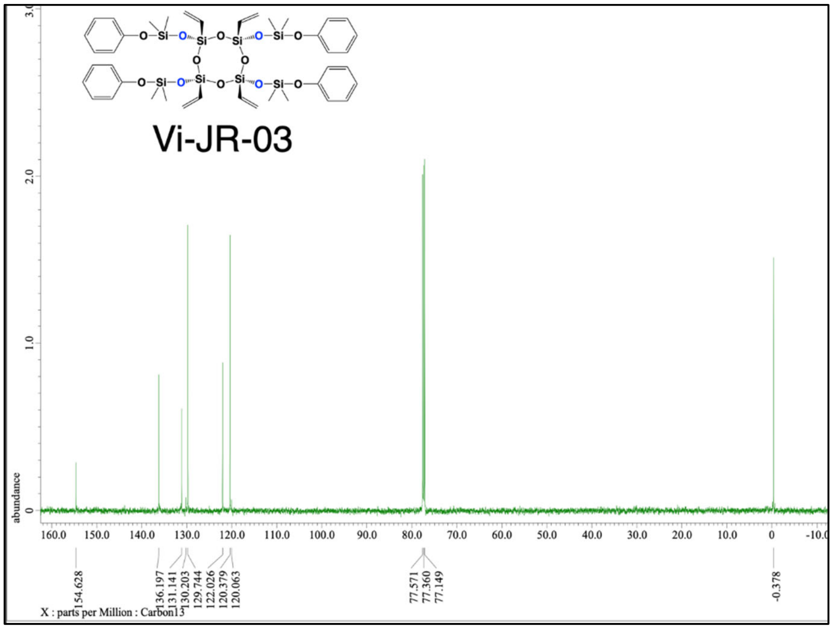

Figure S14. ^{13}C NMR (150.91 MHz, $CDCl_3$): δ –0.38, 120.06, 120.40, 122.03, 129.74, 130.02, 131.41, 136.20, and 154.63 ppm.

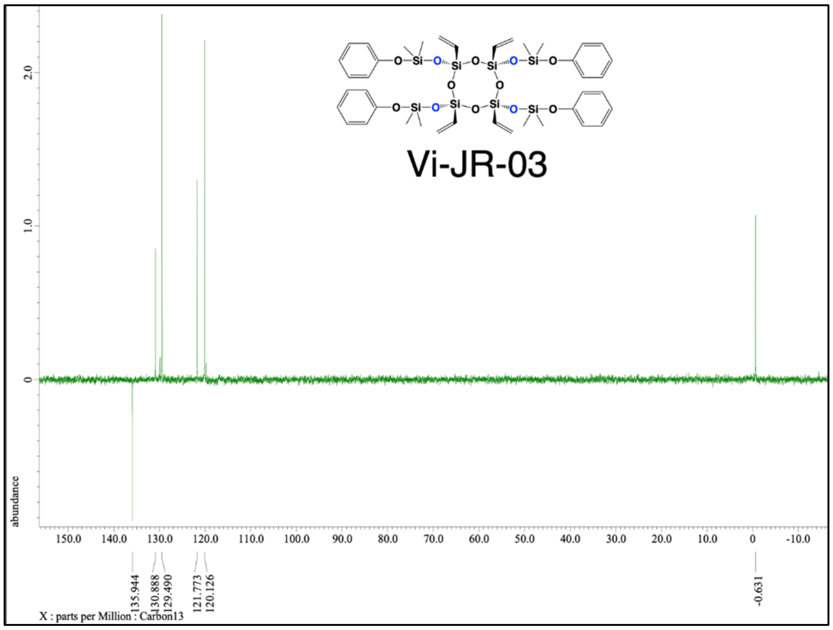

Figure S15. ^{13}C NMR (150.91 MHz, DEPT-135, $CDCl_3$): δ –0.63, 120.13, 121.77, 129.49, 130.89, and 135.94 ppm.

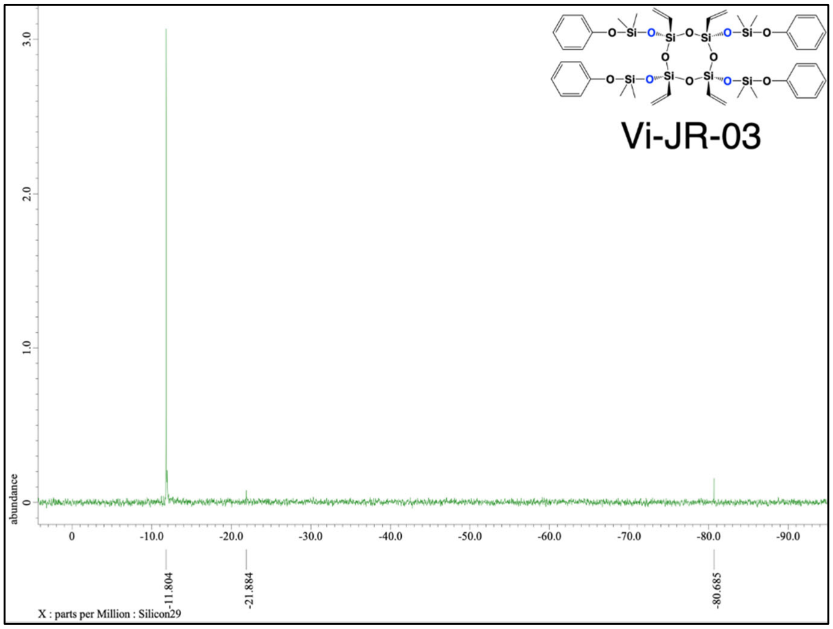

Figure S16. ^{29}Si NMR (119.24 MHz, $CDCl_3$): δ –11.80 ppm (D-unit Si) and –80.69 ppm (T-unit Si).

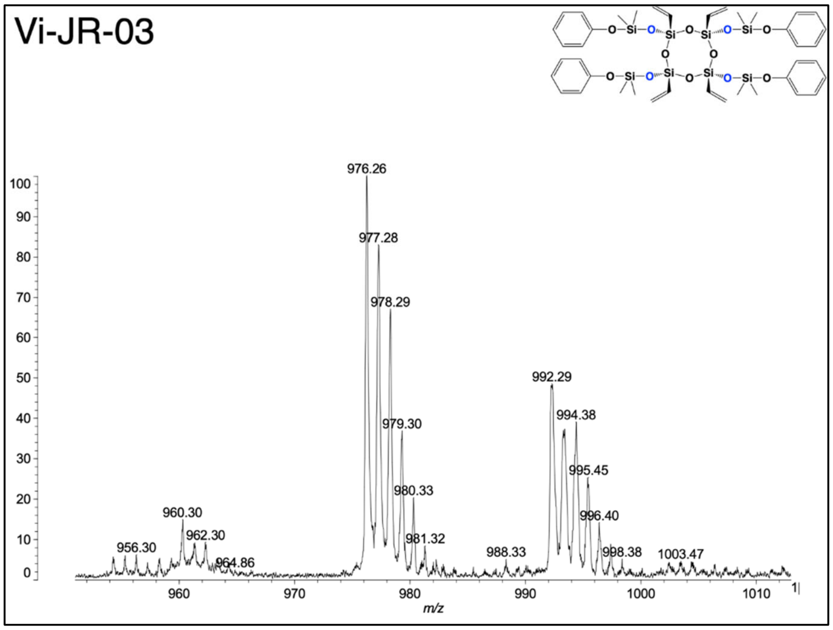

Figure S17. MALDI-TOF results of Vi-JR-03 (Calculated $[M + Na]^+$ = 975.18).

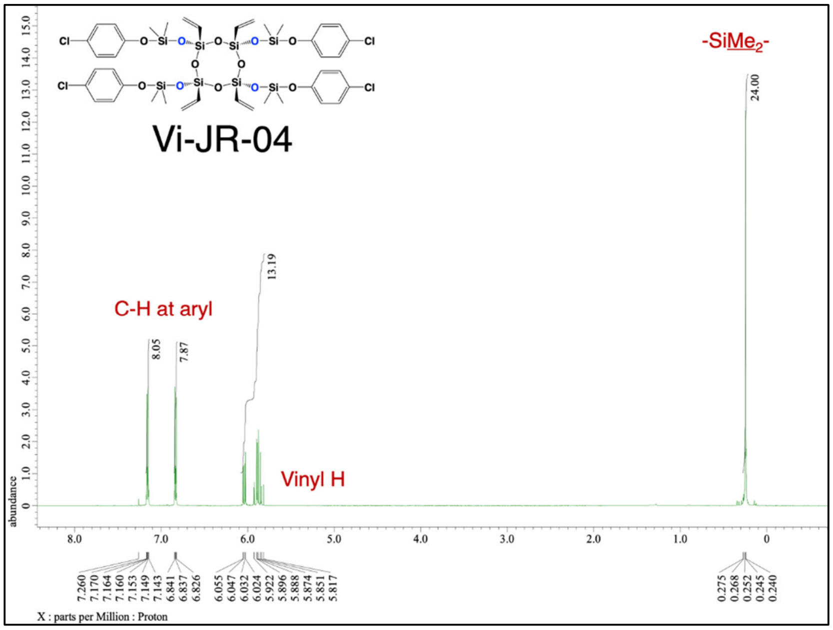

Figure S18. ^{1}H NMR (600.17 MHz, $CDCl_3$): δ 0.25 (s, 24H, Si<u>Me</u>$_2$), 5.82–6.55 (m, 12H, C<u>H</u>=CH_2 and CH=C<u>H</u>$_2$ at vinyl group), and 6.82–7.17 ppm (m, 16H, Ar–H).

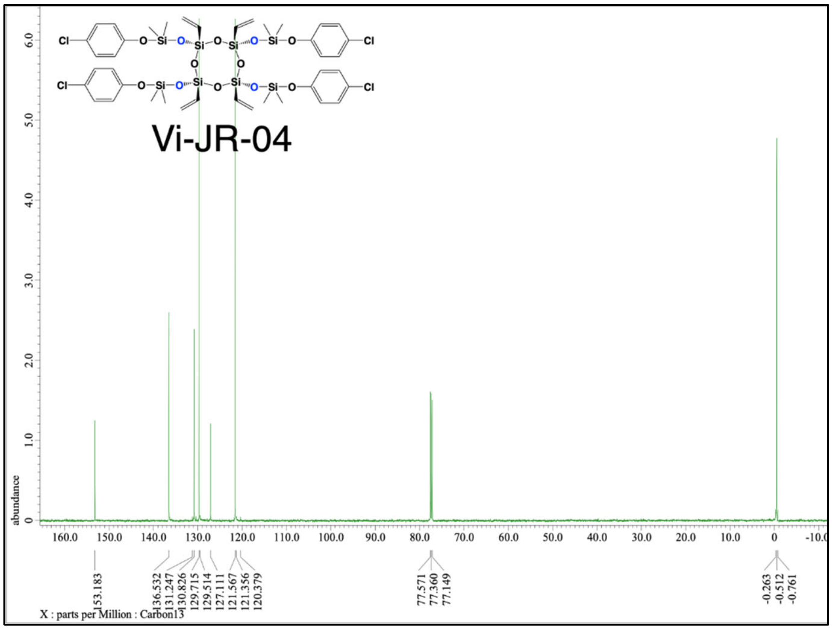

Figure S19. ^{13}C NMR (150.91 MHz, $CDCl_3$): δ –0.51, 120.38, 121.36, 121.57, 127.11, 129.51, 129.72, 130.83, 131.25, 136.53, and 153.18 ppm.

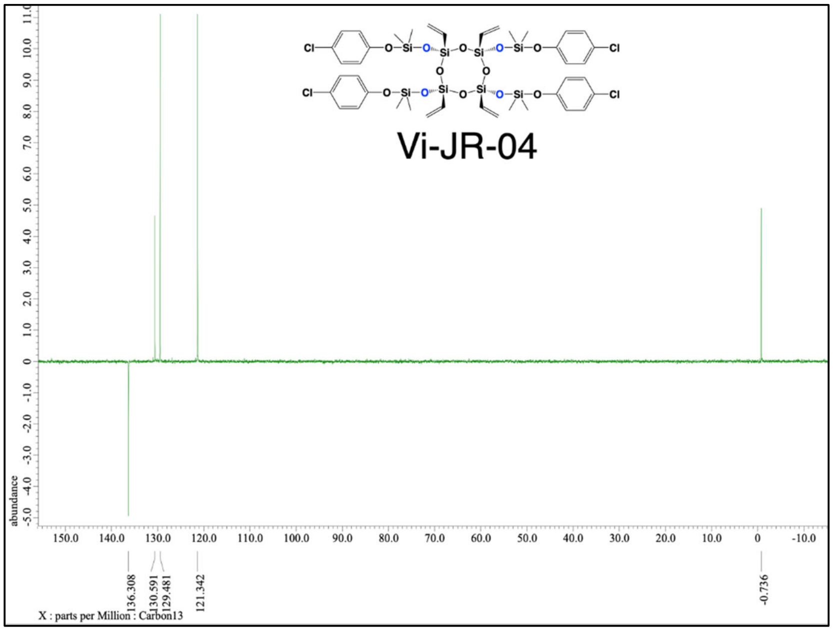

Figure S20. ^{13}C NMR (150.91 MHz, DEPT-135, $CDCl_3$): δ –0.74, 121.34, 129.48, 130.59, and 136.31 ppm.

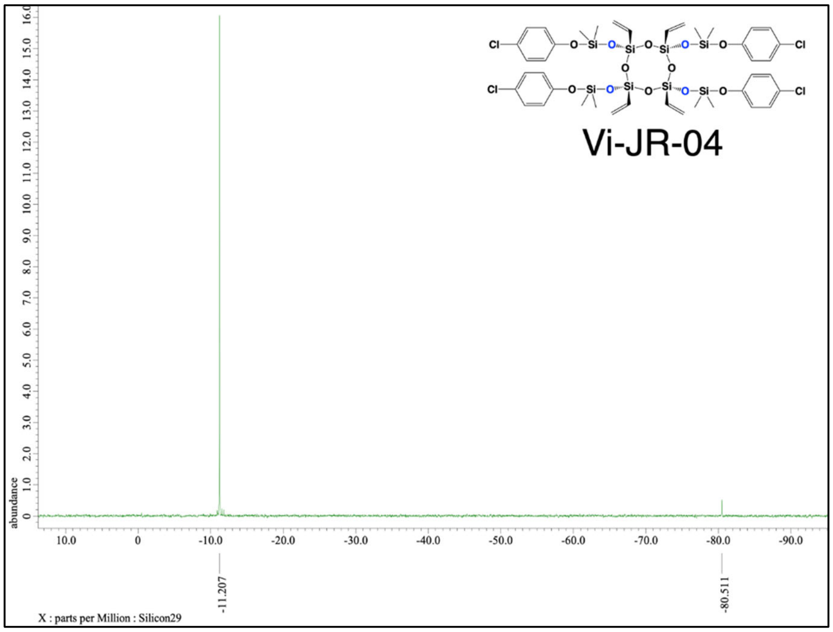

Figure S21. ^{29}Si NMR (119.24 MHz, $CDCl_3$): δ –11.21 ppm (D-unit Si) and –80.51 ppm (T-unit Si).

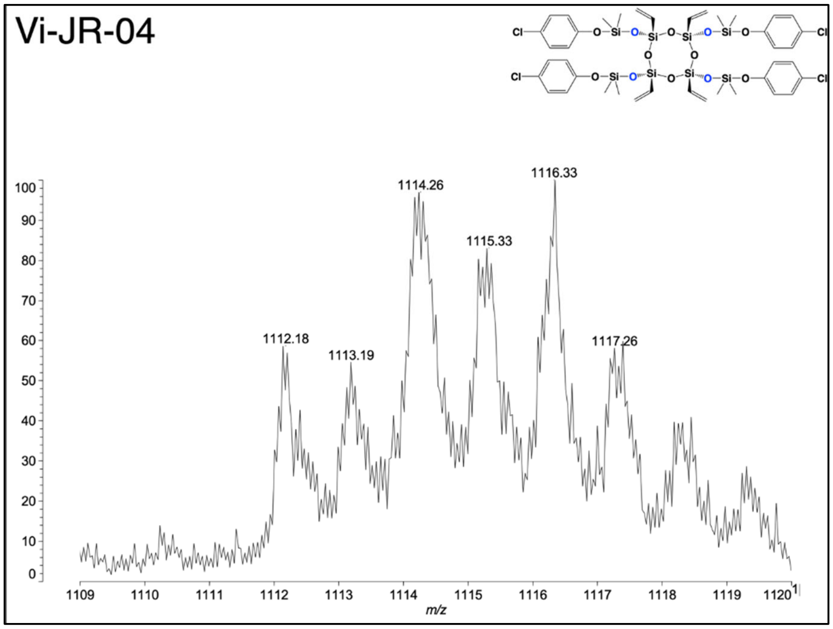

Figure S22. MALDI-TOF results of Vi-JR-04 (Calculated $[M + Na]^+$ = 1113.12).

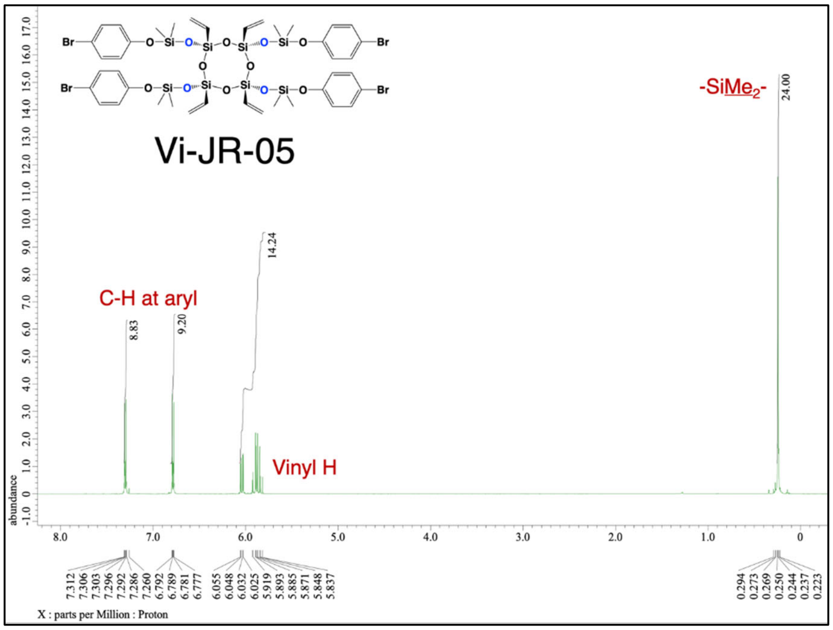

Figure S23. ^{1}H NMR (600.17 MHz, $CDCl_3$): δ 0.25 (s, 24H, Si$\underline{Me}_2$), 5.84–6.55 (m, 12H, C$\underline{H}$=CH_2 and CH=C$\underline{H}_2$ at vinyl group), and 6.78–7.31 ppm (m, 16H, Ar–H).

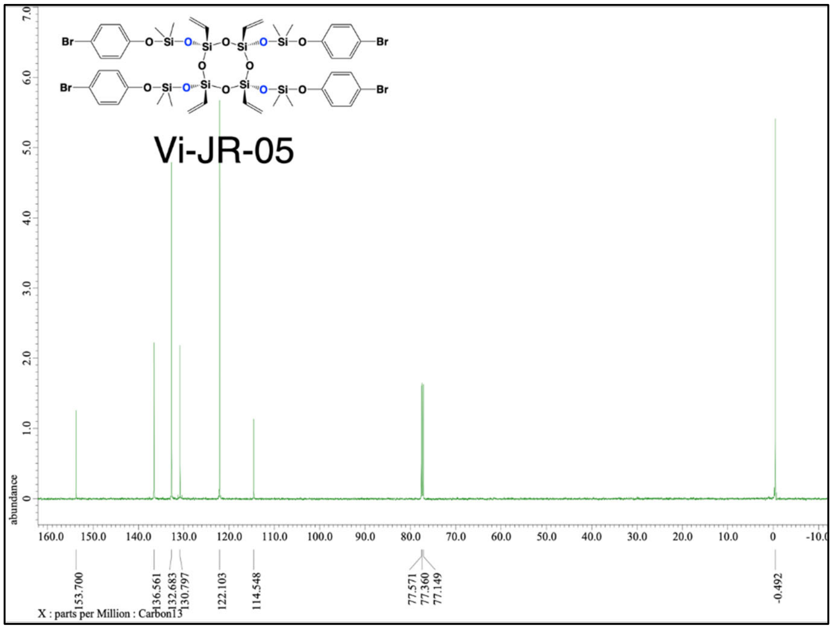

Figure S24. ^{13}C NMR (150.91 MHz, $CDCl_3$): δ –0.49, 114.54, 122.10, 130.98, 132.68, 136.56, and 153.70 ppm.

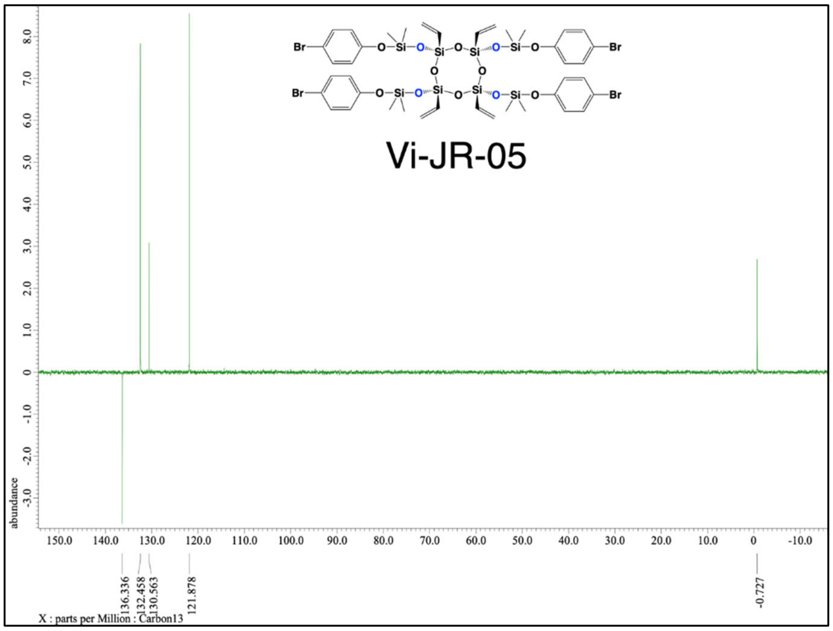

Figure S25. ^{13}C NMR (150.91 MHz, DEPT-135, $CDCl_3$): δ –0.73, 121.88, 130.56, 132.46, and 136.34 ppm.

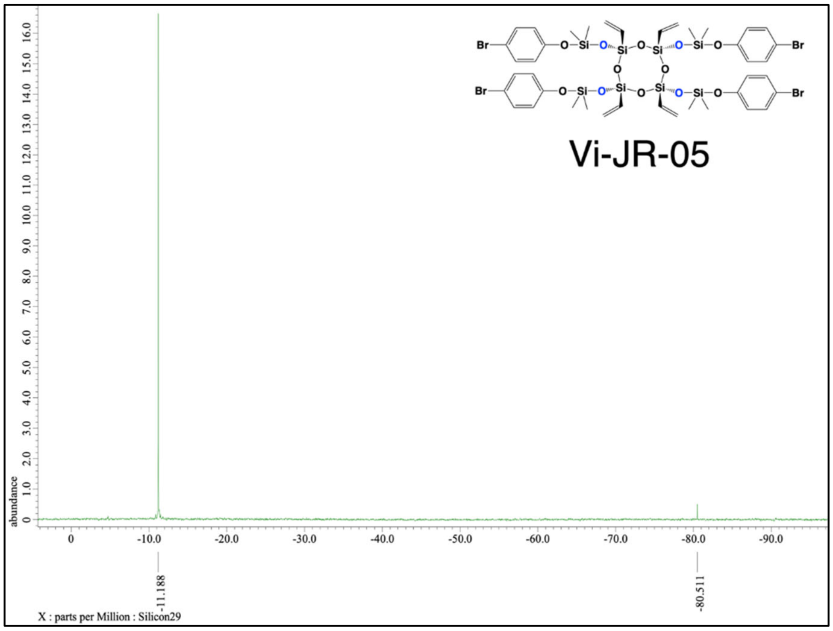

Figure S26. ^{29}Si NMR (119.24 MHz, $CDCl_3$): δ –11.19 ppm (D-unit Si) and –80.51 ppm (T-unit Si).

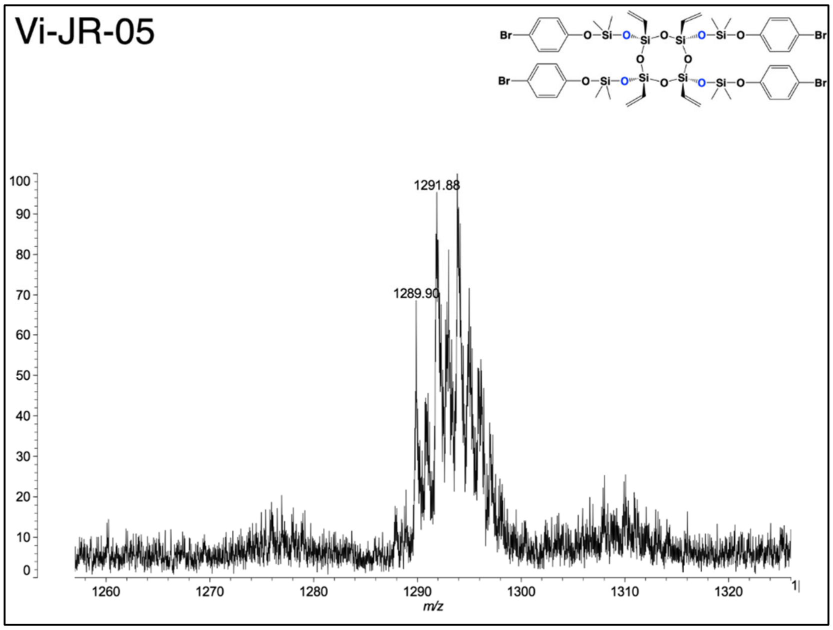

Figure S27. MALDI-TOF results of Vi-JR-05 (Calculated $[M + Na]^+$ = 1290.82).

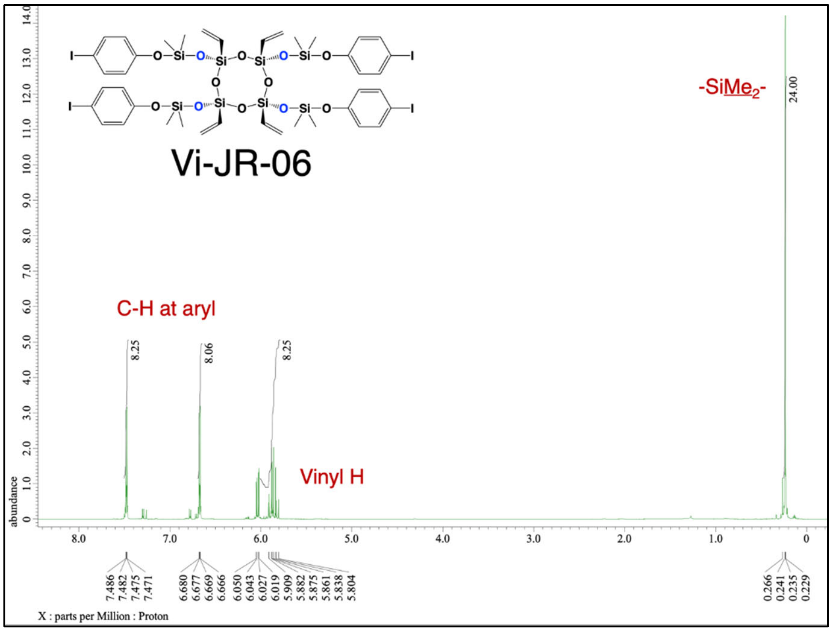

Figure S28. ^{1}H NMR (600.17 MHz, $CDCl_3$): δ 0.24 (s, 24H, Si<u>Me</u>$_2$), 5.80–6.05 (m, 12H, C<u>H</u>=CH_2 and CH=C<u>H</u>$_2$ at vinyl group), and 6.67–7.49 ppm (m, 16H, Ar–H).

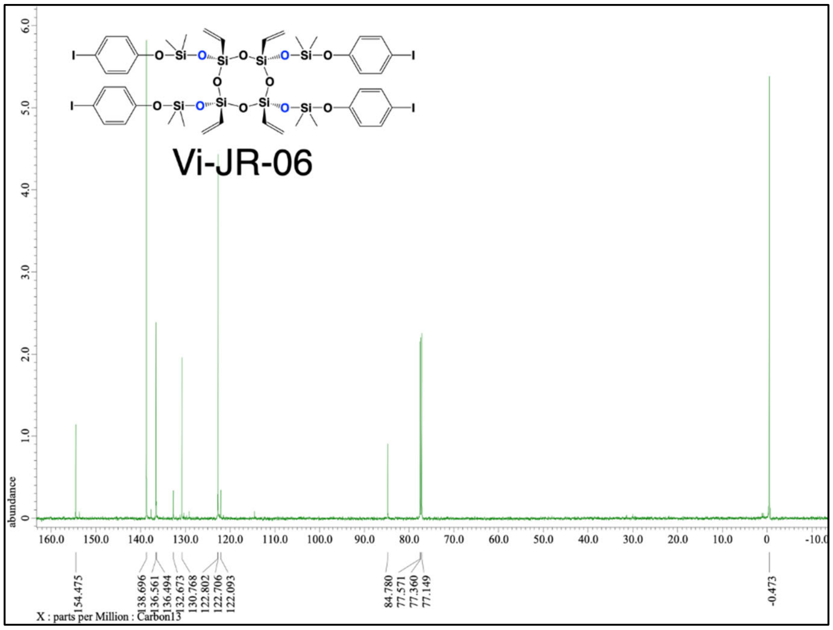

Figure S29. ^{13}C NMR (150.91 MHz, $CDCl_3$): δ –0.47, 84.78, 122.71, 130.77, 136.56, 138.70, and 154.47 ppm.

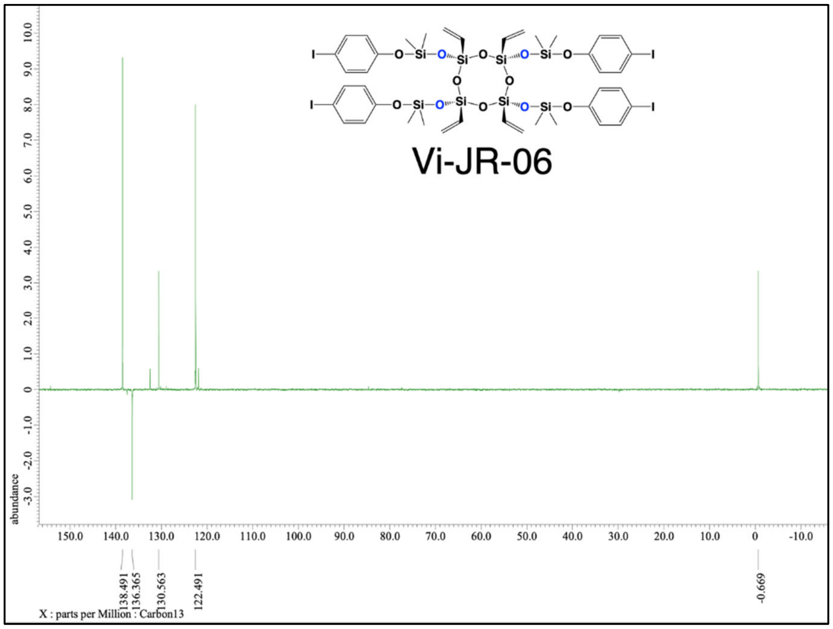

Figure S30. ^{13}C NMR (150.91 MHz, DEPT-135, $CDCl_3$): δ –0.67, 122.49, 130.56, 136.37, and 138.49 ppm.

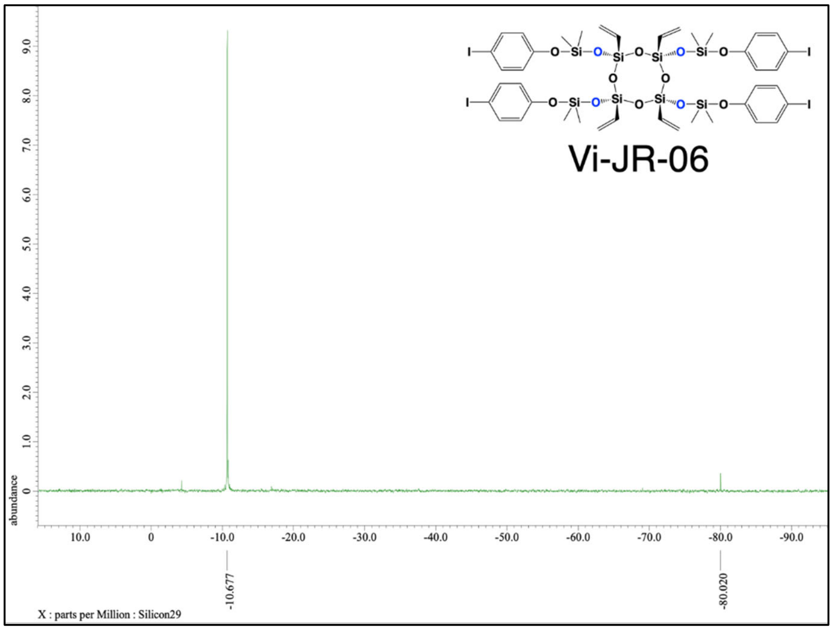

Figure S31. ^{29}Si NMR (119.24 MHz, $CDCl_3$): δ –10.68 ppm (D-unit Si) and –80.02 ppm (T-unit Si).

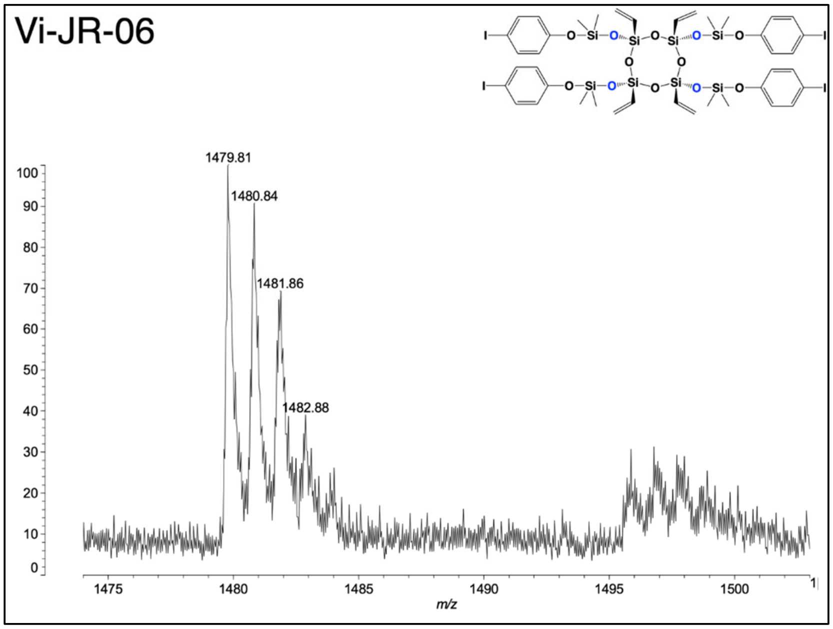

Figure S32. MALDI-TOF results of Vi-JR-06 (Calculated $[M + Na]^+$ = 1478.77).

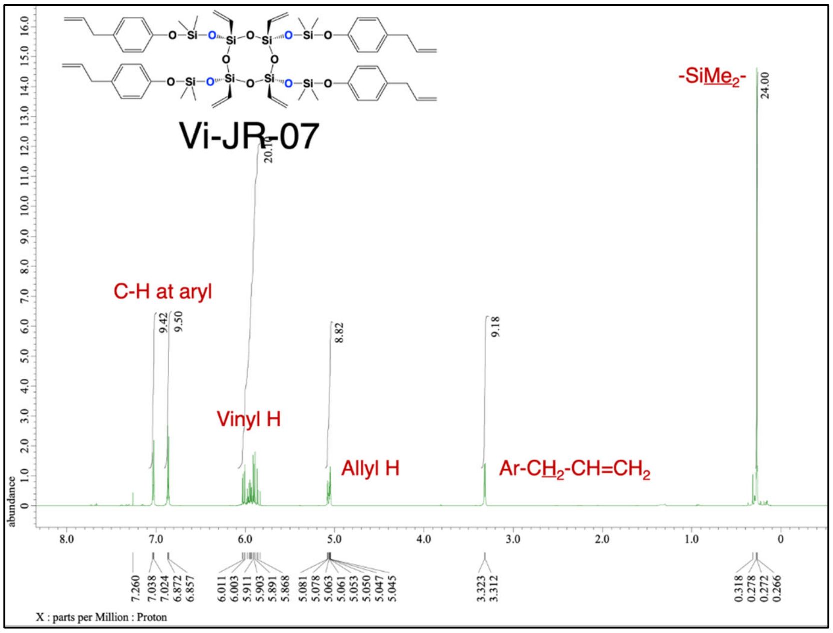

Figure S33. ^{1}H NMR (600.17 MHz, $CDCl_3$): δ 0.27 (s, 24H, $Si\underline{Me}_2$), 3.31–3.32 (d, 8H, Ar–$C\underline{H}_2$–$CH=CH_2$), 5.04–5.08 (m, 8H, Ar–CH_2–$CH=C\underline{H}_2$), 5.87–6.01 (m, 16H, $C\underline{H}=CH_2$, $CH=C\underline{H}_2$, and Ar–CH_2–$CH=C\underline{H}_2$), and 6.85–7.03 ppm (m, 16H, Ar–H).

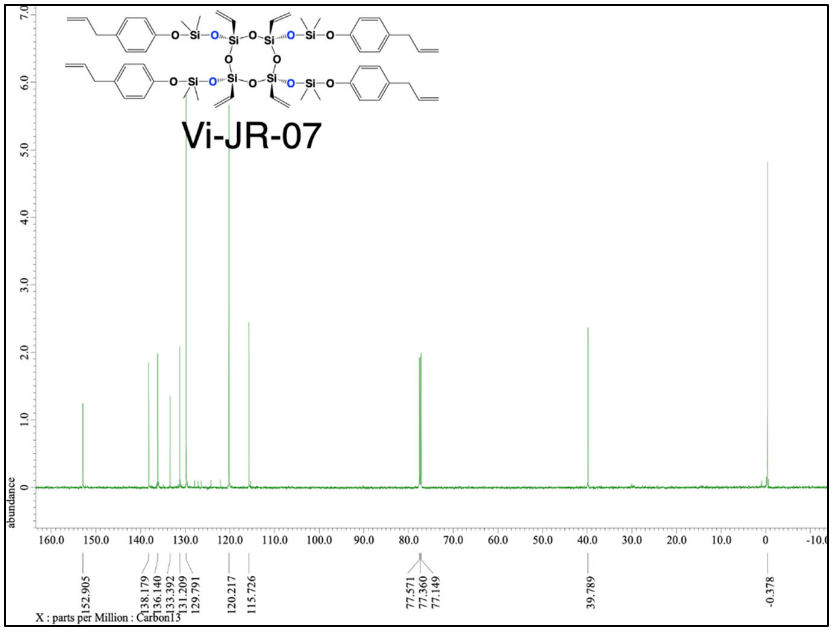

Figure S34. ^{13}C NMR (150.91 MHz, $CDCl_3$): δ –0.38, 39.79, 115.73, 120.22, 129.79, 131.21, 133.39, 136.14, 138.18, and 152.91 ppm.

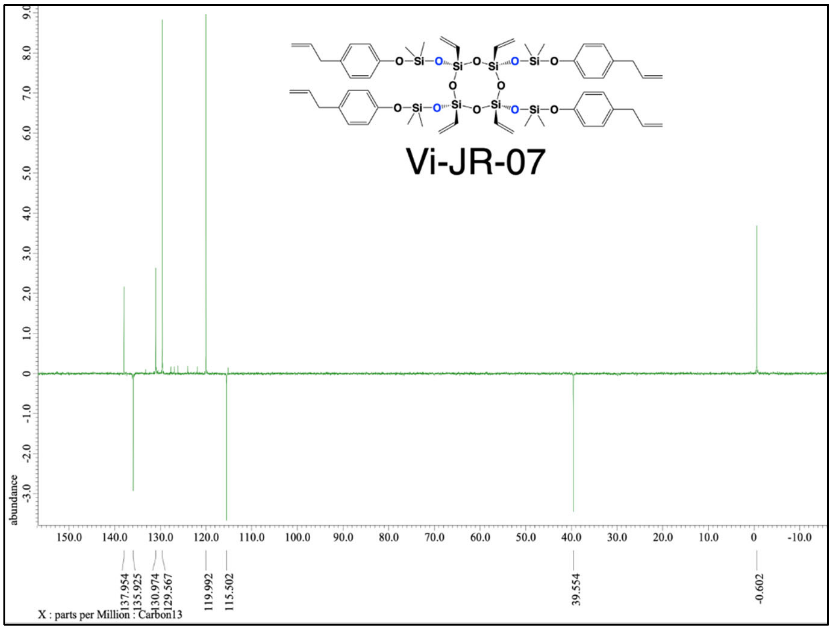

Figure S35. ^{13}C NMR (150.91 MHz, DEPT-135, $CDCl_3$): δ –0.60, 39.55, 115.50, 119.99, 129.57, 130.97, 135.93, and 137.95 ppm.

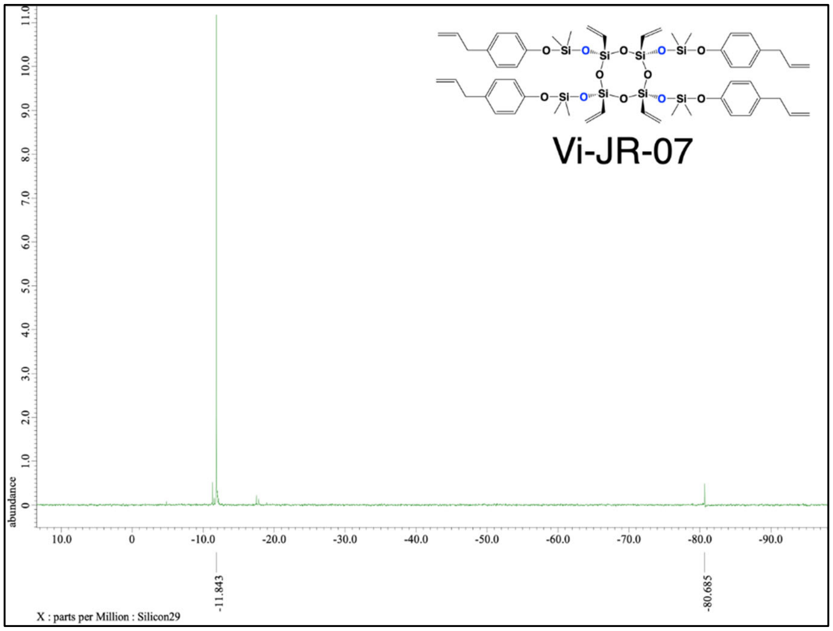

Figure S36. ^{29}Si NMR (119.24 MHz, $CDCl_3$): δ –11.84 ppm (D-unit Si) and –80.68 ppm (T-unit Si).

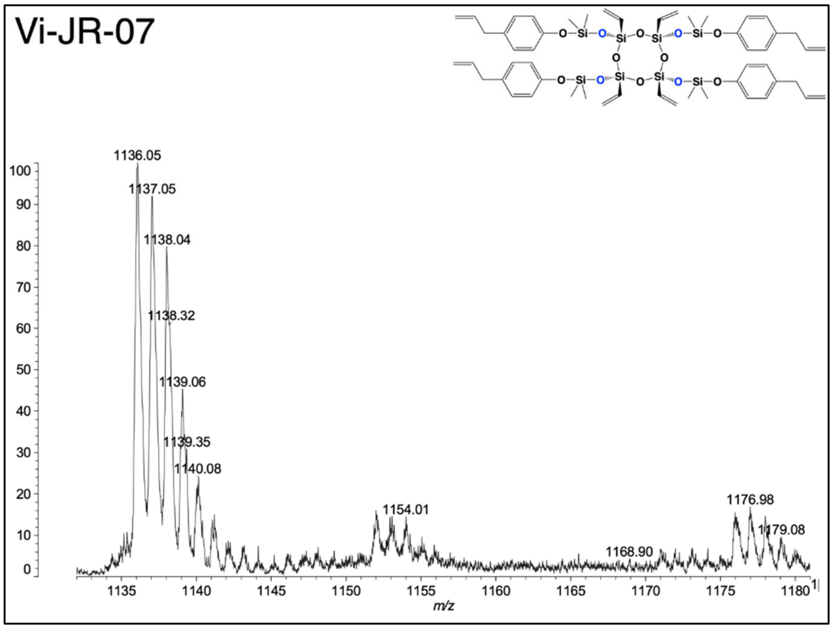

Figure S37. MALDI-TOF results of Vi-JR-07 (Calculated $[M + Na]^+$ = 1135.31).

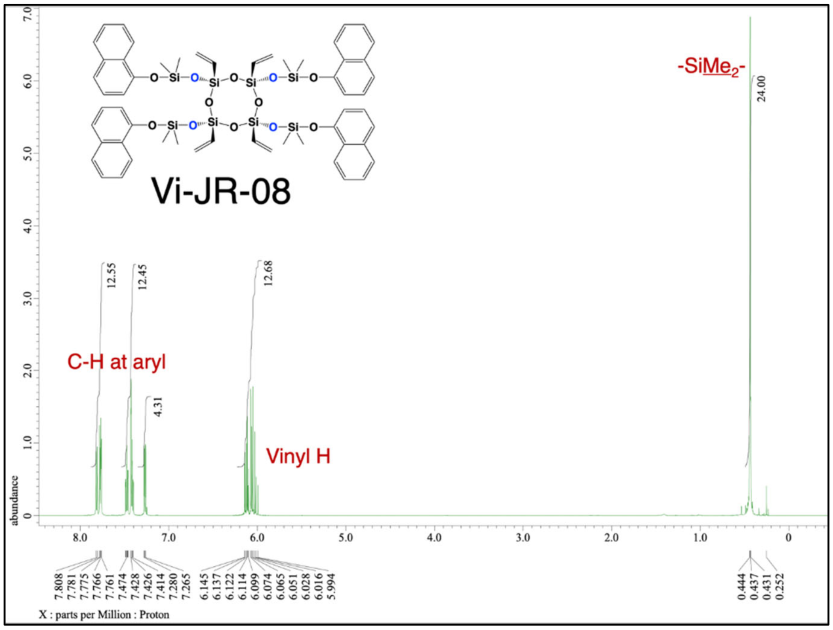

Figure S38. ^{1}H NMR (600.17 MHz, $CDCl_3$): δ 0.44 (s, 24H, Si$\underline{Me}_2$), 5.99–6.15 (m, 12H, C$\underline{H}$=CH_2 and CH=C$\underline{H}_2$ at vinyl group), and 7.28–7.81 ppm (m, 16H, Ar–H).

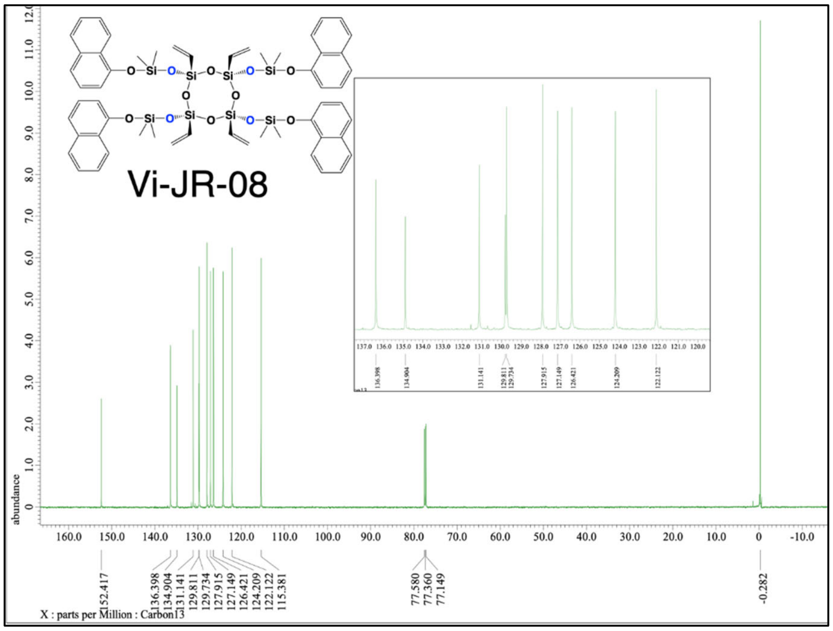

Figure S39. ^{13}C NMR (150.91 MHz, $CDCl_3$): δ –0.28, 115.38, 122.12, 124.21, 126.42, 127.15, 127.92, 129.73, 129.81, 131.41, 134.90, 136.40, and 152.42 ppm.

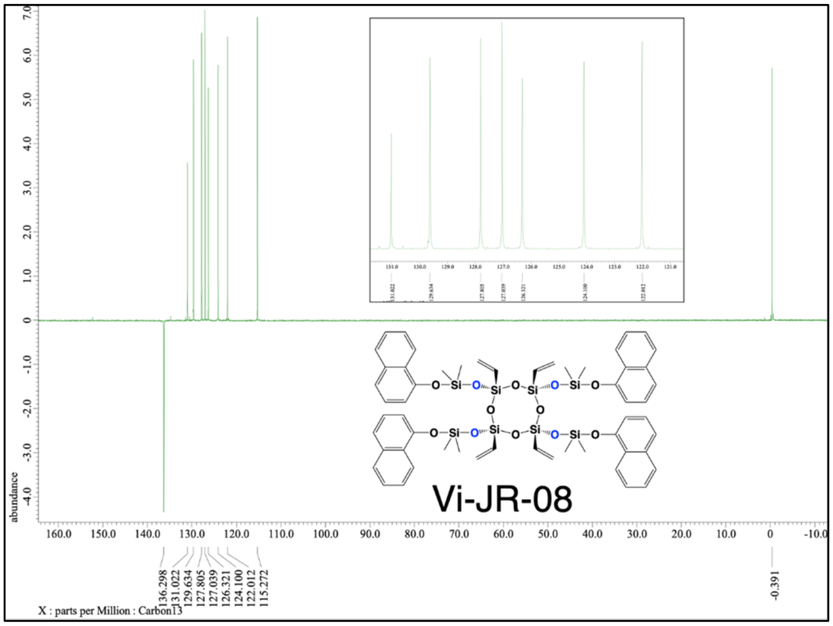

Figure S40. ^{13}C NMR (150.91 MHz, DEPT-135, $CDCl_3$): δ –0.39, 115.27, 122.01, 124.10, 126.32, 127.04, 127.81, 129.63, 131.02, and 136.30 ppm.

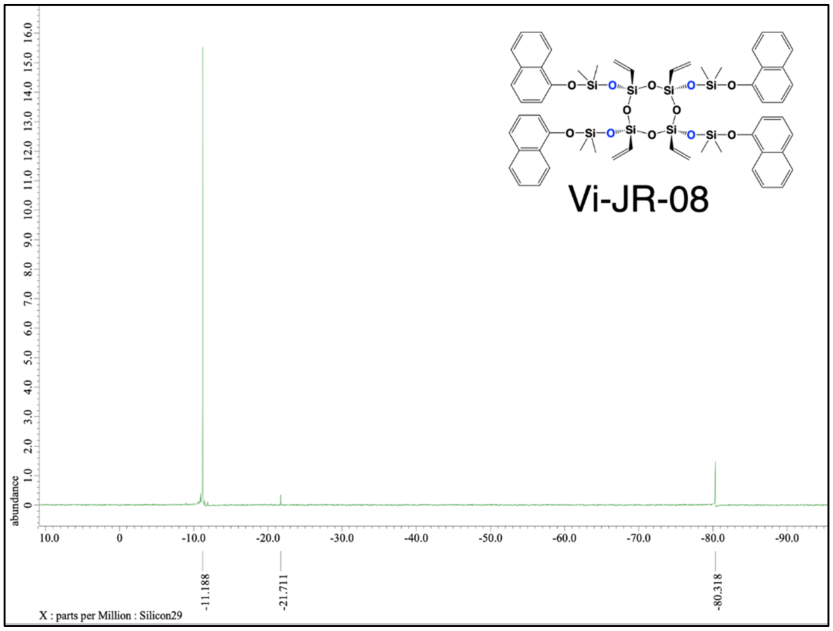

Figure S41. ^{29}Si NMR (119.24 MHz, $CDCl_3$): δ –11.18 ppm (D-unit Si) and –80.32 ppm (T-unit Si).

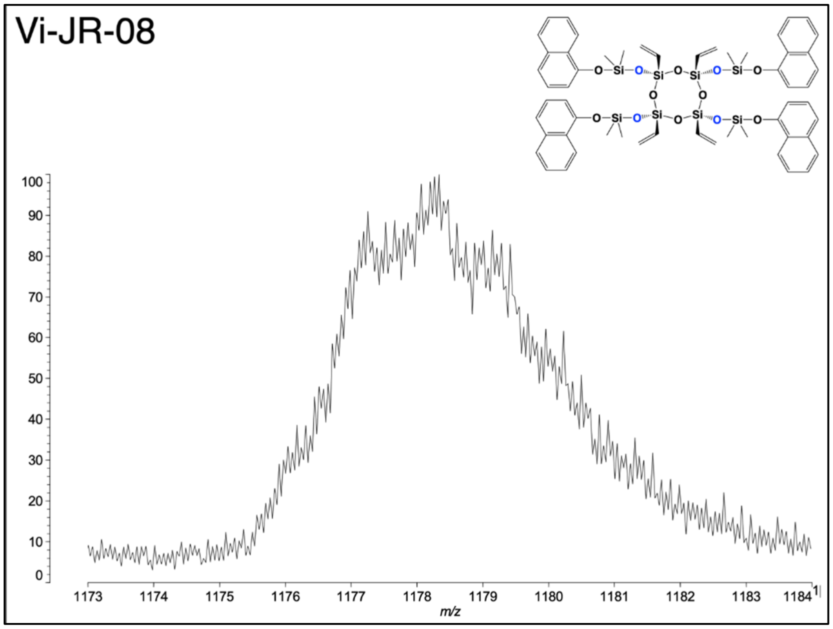

Figure S42. MALDI-TOF results of Vi-JR-08 (Calculated $[M + Na]^+ = 1775.24$).

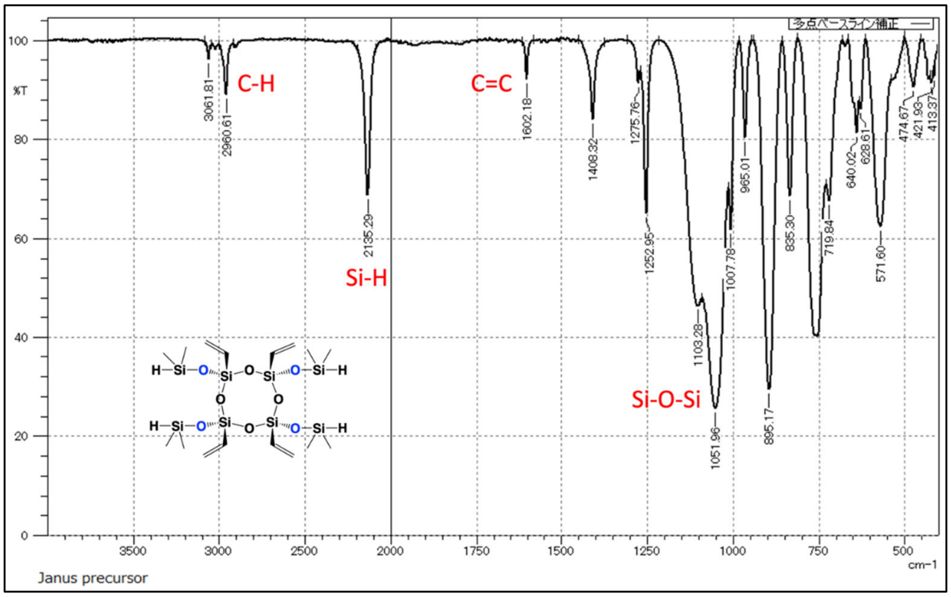

Figure S43. FT-IR result of starting precursor.

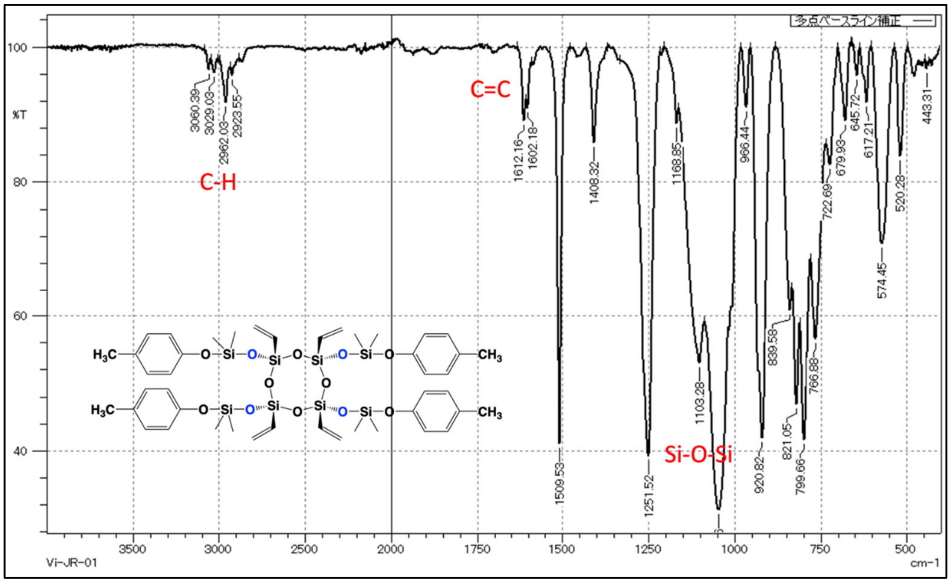

Figure S44. FT-IR result of Vi-JR-01.

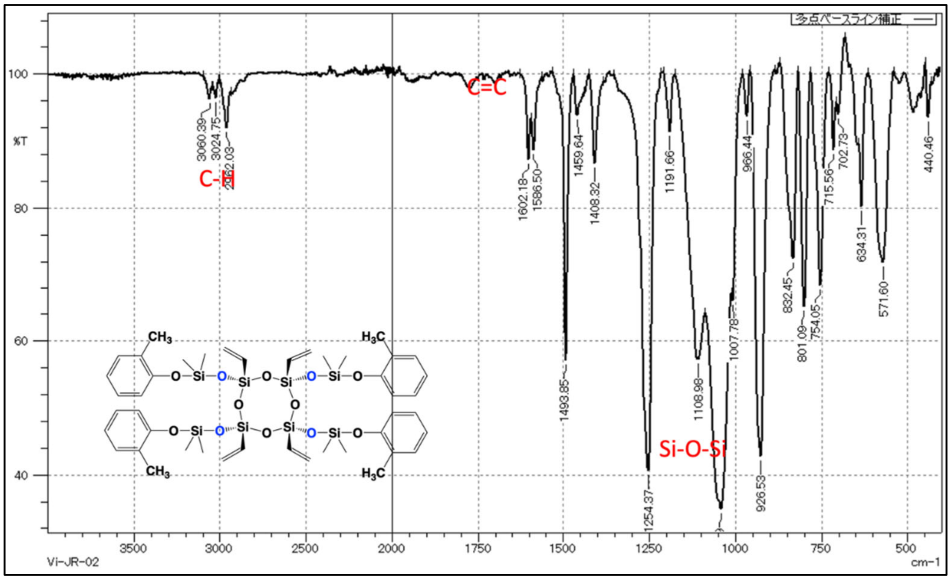

Figure S45. FT-IR result of Vi-JR-02.

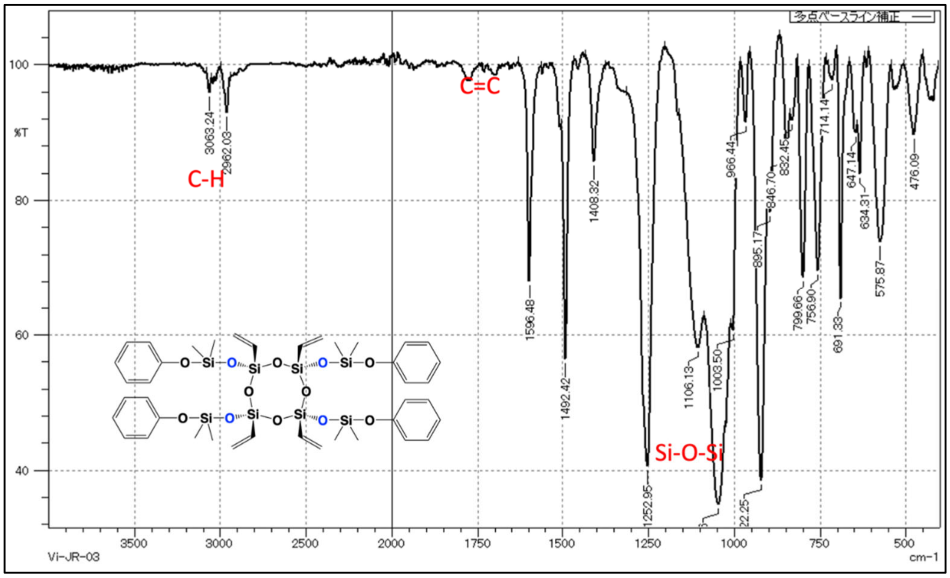

Figure S46. FT-IR result of Vi-JR-03.

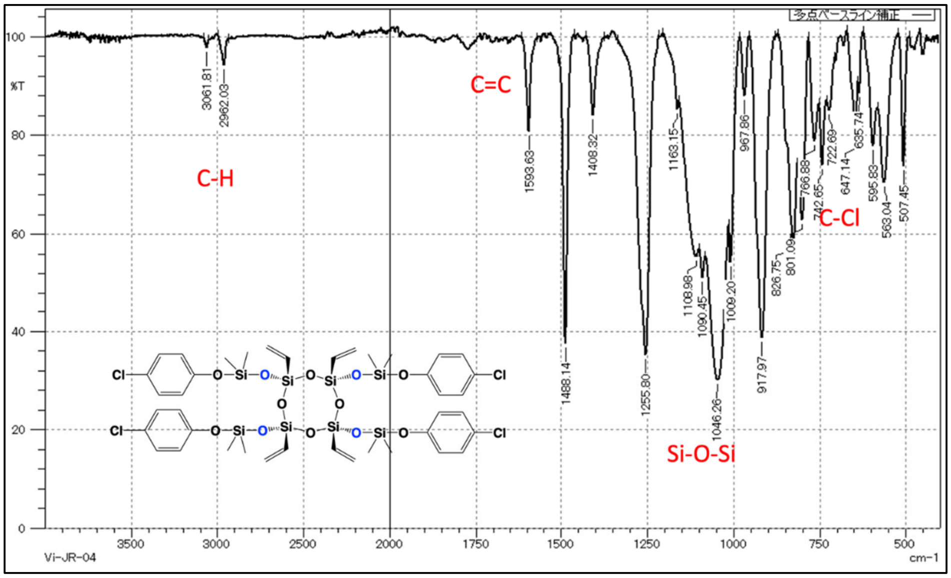

Figure S47. FT-IR result of Vi-JR-04.

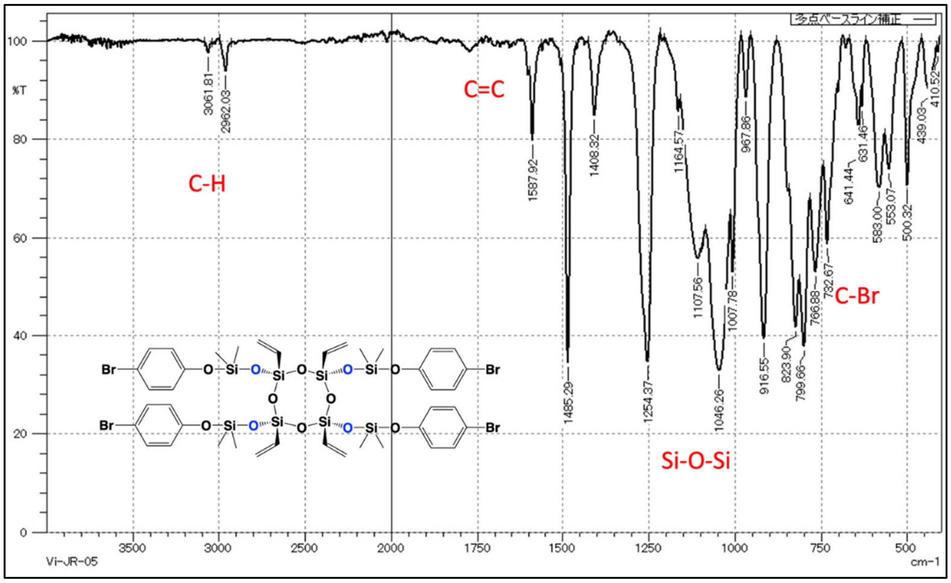

Figure S48. FT-IR result of Vi-JR-05.

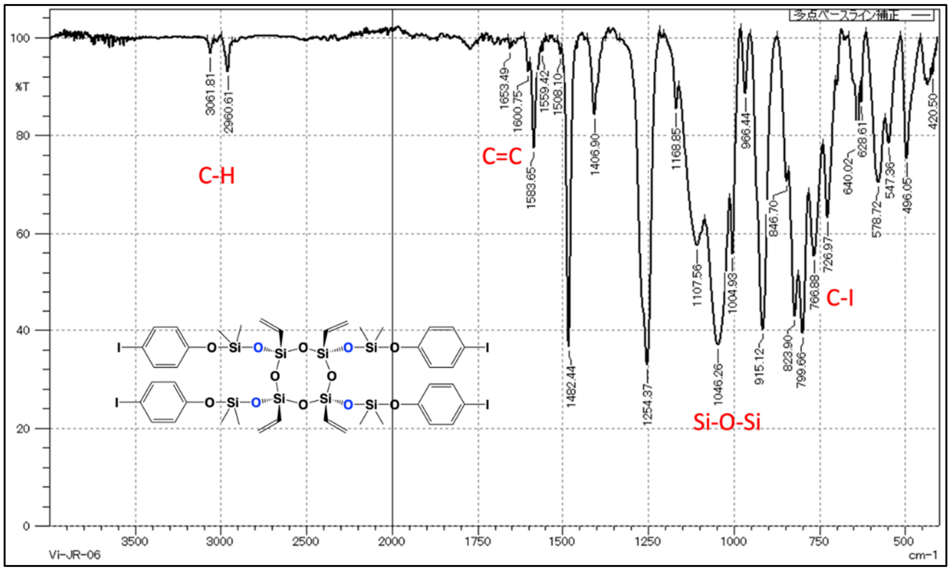

Figure S49. FT-IR result of Vi-JR-06.

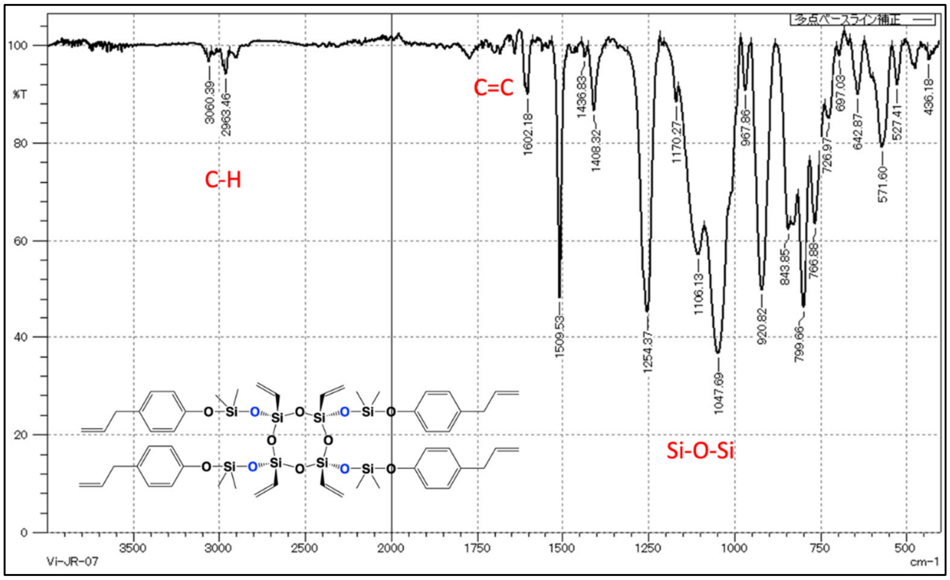

Figure S50. FT-IR result of Vi-JR-07.

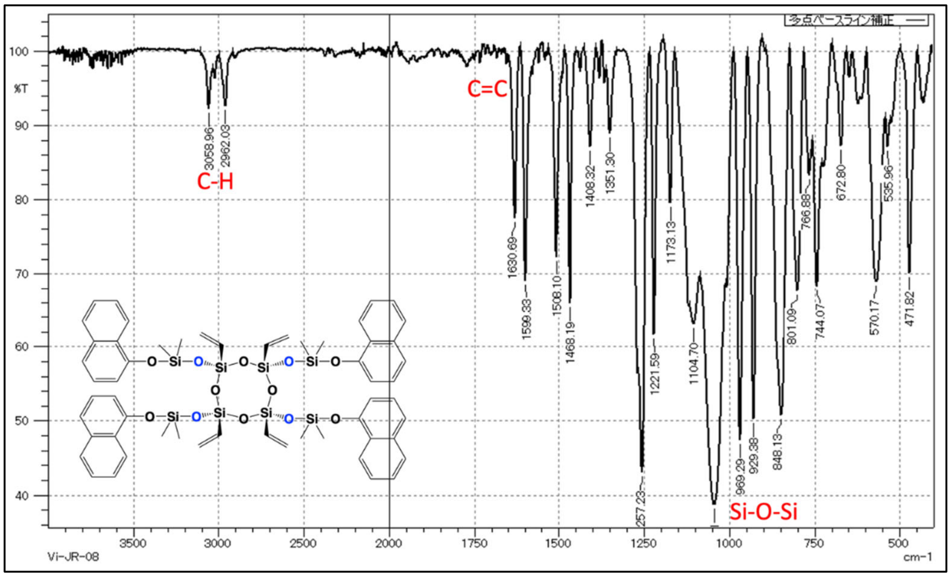

Figure S51. FT-IR result of Vi-JR-08.
